# Artificial Organelles with Digesting Characteristics: Imitating Simplified Lysosome‐ and Macrophage‐Like Functions by Trypsin‐Loaded Polymersomes

**DOI:** 10.1002/advs.202207214

**Published:** 2023-04-19

**Authors:** Xiaoying Xu, Silvia Moreno, Susanne Boye, Peng Wang, Brigitte Voit, Dietmar Appelhans

**Affiliations:** ^1^ Deaprtment Bioactive and Responsive Polymers Leibniz‐Institut für Polymerforschung Dresden e.V. Hohe Straße 6 D‐01069 Dresden Germany; ^2^ Organic Chemistry of Polymers Technische Universität Dresden D‐01062 Dresden Germany; ^3^ Center Macromolecular Structure Analysis Leibniz‐Institut für Polymerforschung Dresden e.V. Hohe Straße 6 D‐01069 Dresden Germany

**Keywords:** artificial organelles, lysosomes, macrophages, polymeric vesicles

## Abstract

Defects in cellular protein/enzyme encoding or even in organelles are responsible for many diseases. For instance, dysfunctional lysosome or macrophage activity results in the unwanted accumulation of biomolecules and pathogens implicated in autoimmune, neurodegenerative, and metabolic disorders. Enzyme replacement therapy (ERT) is a medical treatment that replaces an enzyme that is deficient or absent in the body but suffers from short lifetime of the enzymes. Here, this work proposes the fabrication of two different pH‐responsive and crosslinked trypsin‐loaded polymersomes as protecting enzyme carriers mimicking artificial organelles (AOs). They allow the enzymatic degradation of biomolecules to mimic simplified lysosomal function at acidic pH and macrophage functions at physiological pH. For optimal working of digesting AOs in different environments, pH and salt composition are considered the key parameters, since they define the permeability of the membrane of the polymersomes and the access of model pathogens to the loaded trypsin. Thus, this work demonstrates environmentally controlled biomolecule digestion by trypsin‐loaded polymersomes also under simulated physiological fluids, allowing a prolonged therapeutic window due to protection of the enzyme in the AOs. This enables the application of AOs in the fields of biomimetic therapeutics, specifically in ERT for dysfunctional lysosomal diseases.

## Graphical abstract

A biomimetic contribution to the restoration of cellular or organelle functions by using therapeutic enzymes and to the enhancement of the limited lifetime of naked enzymes. Macrophage‐ and lysosome‐like functions are imitated by the use of trypsin‐loaded polymersomes in the pH range 7.5–6.5 for capturing and digesting enzymes as model pathogens by adjusting membrane properties.

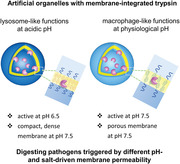

## 1 Introduction

The ultimate objective of developing biomimetic cell structures is to simulate the structural and functional features of cells and their compartments.^[^ ^1^ ^]^ This began more than several decades ago, when the concept of protein and filament interactions with liposomes, liposome permeability, and liposomes as carriers was proposed,^[^ ^2^ ^]^ paving the way for promising and extremely sophisticated applications in artificial organelles and cells.^[^ ^3^ ^]^

Macrophages are responsible for phagocytosis which is the digestion of dead cells, cell debris, and other harmful organisms that play a crucial role in innate and adaptive immune response.^[^ ^4^ ^]^ Dysfunctional macrophages can lead to serious diseases, such as chronic inflammatory, metabolic, or respiratory diseases, and major infections like the human immunodeficiency virus.^[^ ^5^ ^]^ Macrophage morphology and viability have been discovered to be sensitive to environmental pH changes, showing optimal conditions at physiological pH (pH 7.4).^[^ ^6^ ^]^ A similar function is performed by lysosomes. This organelle is capable of autophagy and the removal of damaged structures by cooperating with phagosomes in the phagocytosis of macrophages to break down pathogens. Moreover, dysfunctional lysosomal activity has a significant influence on the biology of aging and age‐related disorders, such as Alzheimer's, Parkinson's, and cardiovascular diseases.^[^ ^7^ ^]^ Lysosomes contain more than 60 different enzymes, and their interior is acidic in the pH range of ≈4.5–5.0.^[^ ^7b^ ^]^

Enzyme replacement therapy (ERT)^[^ ^8^ ^]^ is a medical treatment that replaces an enzyme that is deficient or absent in the body. Treatments based on the catalytic activity of enzymes can restore the correct physiological metabolism.^[^ ^9^ ^]^ However, naked enzymes present some challenges, such as a short in vivo half‐life,^[^ ^10^ ^]^ lack of targeted action, and immune system reactions.^[^ ^11^ ^]^ To compensate for the limited lifetime of naked enzymes, one option in therapeutic treatments is to build artificial cellular structures as protective enzyme carriers that take over biological tasks over a longer time such as imitating the digestive function of macrophages and lysosomes.^[^ ^12^ ^]^ Finding creative and highly sophisticated approaches and solutions with high potential in this regard is a high but challenging goal for scientists.^[^ ^12^, ^13^ ^]^ To address this challenge, a potential approach can be to design artificial organelles (AOs) capable of digesting potential pathogens. Therefore, this work focuses on designing AOs capable of degrading biological material in different environments mimicking the function of lysosomes or macrophages which will be a step forward in the therapy of dysfunctional digestive biological processes.

Controlled polymer syntheses and their self‐assembled structures enable the creation of polymersomes as synthetic counterparts to natural liposomes.^[^ ^14^ ^]^ Polymersomes attract growing attention in the fabrication of biomimetic cell structures and functions and therapeutic nanocarriers.^[^ ^15^ ^]^ Polymersomes are based on the self‐assembly of amphiphilic block copolymers, showing an excellent versatility and mechanical and chemical stability.^[^ ^15^, ^24^ ^]^ Enzymes are integrated into the nanocompartments, establishing nanoreactors with long‐term biological/therapeutic functions mimicking AOs,^[^ ^17^ ^]^ for example, for producing ROS,^[^ ^18^ ^]^ ATP,^[^ ^19^ ^]^ detoxifying superoxide and H_2_O_2_,^[^ ^20^ ^]^ inducing the degradation of proteins/peptides or polysaccharides, or converting other smaller biologically active molecules.^[^ ^21^ ^]^

Over the last decade, significant progress has been made in the selective permeability of polymersomes for low‐molecular‐weight metabolites, drugs and nutrients through manipulations in monomer chemistry,^[^ ^22^ ^]^ the introduction of stimuli‐responsive moieties,^[^ ^23^ ^]^ and the incorporation of channels to form transmembrane proteins such as OmpF^[^ ^21^, ^24^ ^]^ or DNA nanopores^[^ ^25^ ^]^ to finally induce intracellularly driven therapeutic traffic, controlled by pH and metal ion homeostasis^[^ ^26^ ^]^ in cell compartments. Both homeostasis processes are crucial characteristics in biological systems because they regulate the intracellular trafficking of biomolecules via various cellular uptake mechanisms or prohibit the uptake of unwanted pathogens via lysosome trafficking for eventual destruction and/or exocytosis processes.^[^ ^27^ ^]^

**Scheme 1:**
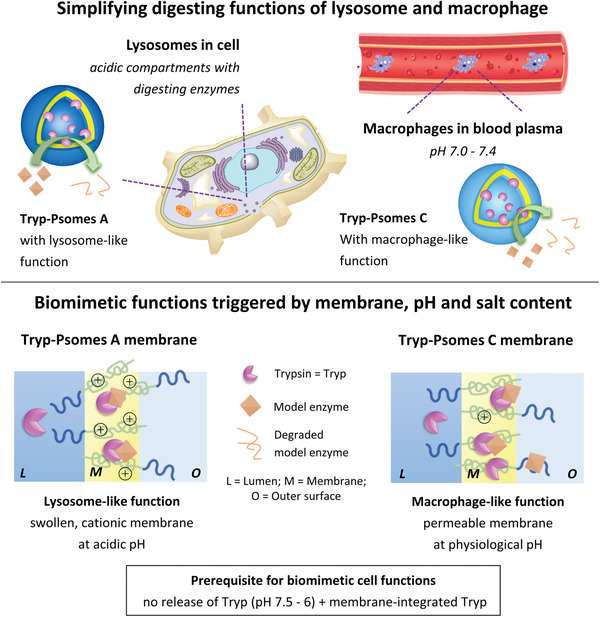
Biomimetic cell functions and structures of lysosome and macrophage through the use of Psomes A and C with lumen‐ and membrane‐integrated Tryp (Tryp‐Psomes A and C) at different environments. Degrading model enzymes of the study are myoglobin and horseradish peroxidase following the concept of i) selective digestion of model pathogens with R ( = arginine) and K ( = lysine) amino acid‐rich units and ii) capturing size‐dependent model pathogens with repellent surface properties of AOs. The membranes of Psomes A and Psomes C have a similar cationic charge state (⊕) under the given experimental conditions. Further details on the digesting and membrane characteristics for biomimetic functions are presented in Figures 4 and 5.

From these results one can conclude, that enzymes are highly adaptable in the location of AOs without losing their enzymatic activity for specific biological and therapeutic applications despite their differences in size, surface charge, and optimum pH. Thus, this versatile platform of stable and highly functional enzyme loaded polymersomes should allow us to establish AOs with selective digestion characteristics based on our pH‐responsive and photo‐crosslinked polymersomes (Psomes)^[^ ^15^, ^23^, ^28^ ^]^ (**Scheme** **1**). The largest hurdle for realizing digesting AOs that capture model pathogens for treatment of dysfunctional lysosomal activity in different environments is to achieve a tunable and size‐selective membrane permeability.^[^ ^29^ ^]^

Previous own results thoroughly outline that Psomes membrane permeability can be smoothly adjusted by the composition of Psomes membrane and salt composition of solutions into the range of physiological pH.^[^ ^15^, ^28^ ^]^ Moreover, pH‐stable membrane‐integrated matrix metalloproetainase‐1 in Psomes is capable of degrading (excess) collagen in an extracellular matrix in vitro and in vivo^[^ ^30^ ^]^ supporting the notion of employing Psomes for the fabrication of novel therapeutic biomimetic cell structures. The selected pH‐responsive Psomes also disclose the desired biocompatibility in in vitro studies^[^ ^30^, ^31^ ^]^ as an important pre‐consideration for the fabrication of digesting AOs.

The goal of this study (Scheme 1) is to mimic simplified lysosomal function at acidic pH and macrophage functions at physiological pH by AOs based on two photo‐crosslinked, pH‐sensitive and trypsin‐loaded Psomes (Tryp‐Psomes; Tryp‐Psomes A; Tryp‐Psomes C). Tryp was selected for integration in digesting AOs (Scheme 1) due to its specific cleavage of R and K amide bonds in biomolecules, while the possible influence of size‐dependent pathogens was investigated by the use of myoglobin (Myo, 17.6 kDa) and horseradish peroxidase (HRP, 44 kDa) as model proteins, which are equipped with K and R amino acid rich units for selective digestion experiments. Overall, the different digestion characteristics of AOs should be thoroughly triggered by pH and ion composition, including the positive or negative ions shielding effect on pathogen´s surface to undergo requested penetration or crossing the membrane of digesting AOs (Figures 4 and 5).

To degrade any pathogens in both biomimetic cell structures, the model pathogens must be in direct contact with Tryp. As a result, it is critical that Tryp is present or diffuses within the membrane (accessible in the swollen and porous state). Already established pH responsive and crosslinked enzyme‐loaded polymersomes promote enzyme activity due to swelling of the membrane at a specific pH which provides control of access of a substrate to the enzyme loaded into the Psomes lumen or inner part of the membrane. This feature can be well used to control digesting of model pathogens at acidic environment mimicking lysozyme function in Tryp‐loaded Psomes (Scheme 1). A semi‐swollen state of the membrane might be preferable since it allows the retention of the loaded Tryp and will provide a size screening of the potential pathogens.

**Figure 1:**
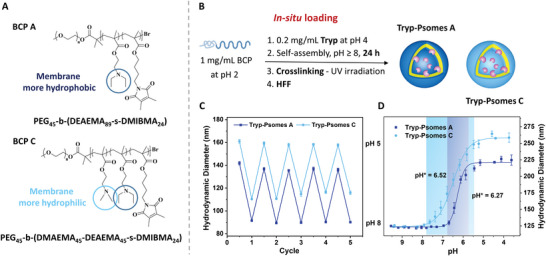
Fabrication and characterization of Tryp‐Psomes by in situ loading: Comparison between both Psomes. A) Chemical structure of BCP‐A and BCP‐C. B) Preparation of Tryp‐Psomes using 180 s as cross‐linking time. C) Size changes by pH switches monitored by DLS of both Tryp‐Psomes. D) pH* comparison by auto titration curve of both Tryp‐Psomes. pH* is 6.27 and 6.52, swelling point is 7.2 and 8.5, respectively. Tryp‐Psomes A and C outline requested permeability for lysosome‐ and macrophage‐like functions as presented in Scheme 1 (bottom). Conditions: 0.5 mg mL^−1^ Tryp‐Psomes in 1 mm PBS (13.7 mm NaCl). HFF = hollow fiber filtration.

Mimicking macrophage‐like digesting and capturing functions (Scheme 1), however, requires a steady enzyme accessibility for model pathogens and enzymatic activity under physiological conditions (pH 7.0–7.5), thus having a constant sufficiently porous Psomes membrane. For that the pH responsivity and suited hydrophilic/hydrophobic balance have to be further adjusted by tuning the block copolymer composition (**Figure** **1**), and Tryp has to be located in the Psomes membrane for easy accessibility for the model pathogens (Scheme 1). The advantage of membrane‐integrated Tryp circumvents the problem of restricted diffusion of large biomolecules with molar masses greater than 40 kDa^[^ ^32^ ^]^ into the lumen of Psomes’.

In addition, the AOs should be applied at different physiological environments (e.g., cytosol, blood plasma or brain) and therefore, the AOs surface should be protein repellent to avoid unspecific protein interactions which limits in vitro and in vivo application.^[^ ^33^ ^]^ By our approach, the therapeutic window of AOs will be expanded from pH 7.5 to pH 6.5, controlled by the environment, and attributed eventually with size‐dependent digestion characteristics (Figures 4 and 5), paving the way for using biomimetic cell structures for further therapeutic applications, for example, in ERT.

## 2 Results and Discussion

### 2.1 Block Copolymers for Artificial Organelles

Two distinct pH‐responsive and crosslinkable block copolymers were produced by atom transfer radical polymerization to acquire broad biological application of Tryp‐Psomes and to control the membrane traffic of Tryp‐Psomes at acidic as well as physiological pH (Figure 1A): i) BCP‐A, hydrophilic poly(ethylene glycol) (PEG) segment and 2‐(*N*,*N′*‐diethylamino)ethyl methacrylate (DEAEMA, pK_B_ 5.2) and 3,4‐dimethylmaleic imidobutyl methacrylate (DMIBMA) as a crosslinker in the hydrophobic segment were used for creating Tryp‐Psomes A with potential lysosome‐like function at acidic pH;^[^ ^23^, ^28^ ^]^ ii) BCP‐C, similar to the previous one, expect that half of DEAEMA was replaced by DMAEMA (2‐(*N*,*N′*‐dimethylamino)ethyl methacrylate),^[^ ^28a^ ^]^ which has a slightly higher pK_B_ value of 5.7, implying protonation at a higher pH, that is, a membrane permeability closer to physiological pH is possible for creating Tryp‐Psomes C with potential macrophage‐like function at physiological pH (Figure 1A). The synthesis and characterization of both BPCs are depicted in Table S1, Figures S1–S8, Supporting Information).

### 2.2 Membrane Characteristics of Artificial Organelles

The working hypothesis of Tryp‐Psomes A and C, which have the potential for preferred cell‐like functions (Scheme 1), respectively, originates from an initial consideration of the different permeability profiles (Figure 1D). This issue will be further verified by the presentation of enzymatic degradation results for both Tryp‐Psomes (Figure 3 and Table 2).

Using a pH switch approach and exposing the formed Psomes to UV light to induce photodimerization of the crosslinker in the hydrophobic Psomes membrane at a basic pH, the self‐assembly of BCP‐A and ‐C resulted in pH‐sensitive and crosslinked Psomes A and C (Empty‐Psomes). The physio‐chemical properties (morphology of vesicles and their membrane, reversible swelling‐shrinking properties, pH responsiveness, etc.) of Empty‐Psomes were investigated (Figures S9–S11, Supporting Information) and compared to Tryp‐Psomes A and C (Figures 1B–D). The capacity of Empty‐Psomes and Enzyme‐Psomes to modulate the pH* value (the turning point at the transition between swelling (dropping pH) and shrinking (raising pH) states) has previously been examined using variations in BCP composition and in the presence of varying salt concentrations.^[^ ^15^, ^28^, ^30^, ^32^, ^34^ ^]^ To fabricate Tryp‐Psomes C as digesting AOs we selected a higher hydrophilic content of DMAEMA in BCP‐C to achieve higher pH* close to physiological pH compared to previously published BCP‐C, possessing a lower pH* as requested for our scientific goal (Scheme 1).^[^ ^28a^ ^]^

#### 2.2.1 Membrane Characteristics of Psomes A and C

For Empty‐Psomes A, as observed in a previous study, pH* values shift close to physiological pH values with increasing salt concentration (pH*≈6.4 (10 mm NaCl), pH*≈6.9 (1 mm PBS), and pH*≈7.2 (10 mm PBS)).^[^ ^15^, ^28^ ^]^ In the present study a similar pH‐ and salt‐responsiveness were also expected for Empty‐Psomes C. pH‐ and salt‐ responsiveness and pH* value were studied in 1 and 10 mm PBS in the presence and absence of 10 mm NaCl for Empty‐Psomes C (**Table** **1**, Figure S10, Supporting Information), possessing (slightly) higher pH* values than those of Psomes A.^[^ ^28a^ ^]^ A larger influence of 10 mm NaCl in 1 mm PBS buffer is observed for Empty‐Psomes C (Figure S10B, Supporting Information). The pH* is 7.07 and 6.86 in 1 mm PBS in the presence and absence of 10 mm NaCl, respectively, proving salt concentration dependency. In the presence of 10 mm PBS with or without 10 mm NaCl, the pH* value is close to the desired physiological pH in both cases (Table 1 and Figure S10A, Supporting Information). Moreover, in comparison with Empty‐Psomes A (pH* = 6.74), Empty‐Psomes C (pH* = 7.54) in 1 mm PBS (with an additional 13.7 mm NaCl) is still semi‐permeable or semi‐swollen at physiological pH (Figure S11, Supporting Information). This key characteristic of Empty‐Psomes C is triggered by the incorporation of 50 mol% hydrophilic monomer DMAEMA in hydrophobic block of BCP‐ C as first explanation (further details later), at which a very low degree of protonated DMAEMA is also assumed in Psomes C membrane due to pK_B_ value of 5.7 for DMAEMA monomer. We hypothesize that the less hydrophobic membrane of Empty‐Psomes C with increased DMAEMA content (Table 1) is even more NaCl‐ and proton‐sensitive compared to Empty‐Psomes A membrane. This is probably caused by the Na^+^ ions interaction with the tertiary amino groups. Therefore, larger membrane hydrophilicity is provided which implies an enhanced permeability toward H^+^ as well as other ions. Finally, a pH* shift is initiated to neutral or slightly basic conditions.^[^ ^28a^ ^]^ Overall, the membrane characteristics of Empty‐Psomes C open the possibility to fabricate Tryp‐Psomes C (Figure 1B) with enzymatic activity ( = macrophage‐like digestion function) at physiological pH.

**Table 1:** The pH* and swelling point of Psomes C (self‐assembly by BCP‐C: PEG_45_‐b‐(DEAEMA_45_ *‐co‐*DMAEMA_45_ *‐co‐*DMIBMA_24_), 1 mg mL^−1^, crosslinking 3 min) in different conditions

Conditions	pH*	Swelling point
10 mm PBS with 10 mm NaCl	7.51	8.54
10 mm PBS without 10 mm NaCl	7.43	8.57
1 mm PBS with 10 mm NaCl	7.07	7.93
1 mm PBS without 10 mm NaCl	6.86	7.97
1 mm PBS with 13.7 mm NaCl – Autotitration	7.54	8.55

#### 2.2.2 Membrane Characteristics of Tryp‐Psomes A and C

The next following step was to load Tryp (0.2 mg mL^−1^) in both Psomes (1.0 mg mL^−1^) by the in situ loading method for 24 h at pH 8. Afterward, the samples were UV light irradiated (180 s) and purified by HFF in 1 mm PBS at pH 7.5 (Figure 1B). Stability and activity studies were performed for free Tryp (Figures S12–S14, Table S4, Supporting Information), and pH 7.5 was validated as the optimum pH for long‐term use (≥1 day). Thus, it was verified that the enzyme activity (further details are presented below) was maintained during the fabrication process (Figure 1B). Cyclic pH switches of Tryp‐Psomes A and C were performed at pH 8.0 (collapsed state) and 5.0 (swollen state) for 5 cycles also proving no disassembly and membrane rupture as well as no aggregation with increasing ion concentrations (Figure 1C).

The pH* values in 1 mm PBS are 6.27 and 6.52 for HFF‐purified Tryp‐Psomes A and Tryp‐Psomes C, respectively (Figure 1D). Tryp‐Psomes A show a sharper transition state, while at pH ≥ 7 Tryp‐Psomes A membrane is practically closed and in collapsed state. However, in the case of Tryp‐Psomes C, its membrane begins to open at pH ≤ 8.5, and remains in a semi‐permeable state until pH 6.5. The integration of Tryp in Psomes C membrane results in the drop of pH* from 7.54 for Empty‐Psomes (Table 1) to 6.52 for Tryp‐Psomes C (Figure 1D), while there is no real change in the swelling point compared to Empty‐Psomes C. Based on this result, it can be predicted that in a pH range of 6–7.5, biomolecules can diffuse into the lumen/membrane and be degraded in both Tryp‐Psomes. However, only Tryp‐Psomes C will be active or permeable around physiological pH (≥7) due to the specific membrane characteristics.

### 2.3 Enzymatic Activity of Free Trypsin and Trypsin in Artificial Organelles

To establish active Tryp‐Psomes with lysosome‐ and macrophage‐like capturing and digesting functions (Scheme 1), it is necessary to understand the reactivity and stability of free Tryp prior, responsible for the cleavage of amide bonds of lysine (K, R^1^) and arginine (R, R^1^) in pathogens (Figure 3C). Briefly summarizing the activity and stability of free Tryp (Figures S12–S14, Supporting Information), free Tryp is more active at pH 8 (Figure S13A, Supporting Information). However, it is more stable at pH 6 (Figure S13B, Supporting Information), because a long incubation period at pH 8 compromises its activity. For the long‐term use of Tryp‐Psomes C with macrophage‐like functions at physiological pH, free Tryp outlines the desired enzymatic stability over several days in 1 mm PBS at pH 7.4 (Figure S14, Supporting Information).

We intended to investigate the desirable enzymatic activity and stability of Tryp‐Psomes for the rapid usage of Tryp‐Psomes following HFF purification using our knowledge of the pH‐ and time‐dependent activity of free Tryp (Figures S12–S14, Supporting Information). The enzyme activity of Tryp‐Psomes A (Figure S15, Supporting Information) is similar to that of free Tryp (Figure S13A, Supporting Information), using a typical Tryp assay. The lowest activity with swollen Psomes membranes after 1 h is found at pH 6. Thus, the Tryp activity is regulated by the pH and less by the accessibility of Tryp through the swollen membrane. Surprisingly, the small substrate of the assay is capable of undergoing Tryp conversion in Tryp‐Psomes A even in the collapsed state (pH 8), also indicating the presence of membrane‐integrated Tryp in the Psomes membrane.

Based on these findings, Tryp activity for Tryp‐Psomes A and C was investigated after 1 day at pH 6.5 and pH 7.5 (Figure S16, Supporting Information), indicating distinct membrane states: closed versus opened for Tryp‐Psomes A or steadily permeable for Tryp‐Psomes C. Overall the enzyme activity is similar in all cases, indicating a competition between pH and membrane permeability on Tryp activity over time. This allowed us to regulate the membrane permeability for the degradation of larger model pathogens in later experiments.

### 2.4 Loading Efficiency, Non‐Releasing Characteristics, and Stability of Trypsin in Artificial Organelles

Before studying the degradation ability, it is important to study whether the Tryp remains loaded in the Psomes, especially when it is preferentially integrated into the Psomes membrane (below more details), or will be released, which will reduce the activity of the artificial organelles over time. This study is crucial for the enzyme Tryp which has a small size (23.3 kDa). From own study we know that the molecular‐weight‐cut‐off of Psomes A membrane in the swollen state is ≈40 kDa.^[^ ^32^ ^]^ To calculate the loading efficiency (LE), Tryp‐Psomes using Rhodamine B labeled Tryp (RhB‐Tryp) were prepared (Figure S17, Supporting Information) and the fluorescence intensity before (not shown) and after (Figure S18, Supporting Information) HFF purification was measured. The LEs are 28.5% and 26.3% for RhB‐Tryp‐Psomes A and RhB‐Tryp‐Psomes C, respectively. To check the release behavior, the purified RhB‐Tryp‐Psomes (reference) were dialyzed against 1 mm PBS buffer at pH 7.5 for 24 h, then 1 mm PBS buffer at pH 7 for 24 h, and finally 1 mm PBS buffer at pH 6 for 24 h (Figure S18, Supporting Information). After the dialysis process in really excess supernatant solution (2 L), LE decreases ≈9%–11% in both cases. The dialysis process might help to break the physical interactions between Tryp and the surface or membrane of Psomes. However, there is no significant change of LE adjusting the pH, for Tryp‐Psomes A it remains ≈19% and for Tryp‐Psomes C ≈16%. This confirms the stability of both Tryp‐Psomes with no obvious enzyme release even in an opened state at pH 6. The RhB groups bounded to Tryp might influence the loading process and further interactions as well (Figure S17, Supporting Information). Besides, LEs determined by enzyme activity of unlabeled Tryp‐Psomes A and Tryp‐Psomes C are 9.26% and 9.02%, respectively, after HFF purification step (Figure S19, Supporting Information). We might indicate that the achieved LE of Tryp (23 kDa) is in the upper range for enzymes with molecular weights of ≈20 kDa (e.g., Myo, 5–10%), independent of used pH‐responsive Psomes for enzyme in situ loading process.^[^ ^34^, ^35^ ^]^

From a therapeutic point of view, it is important to determine the best storage method to ensure adequate reproducibility. HFF‐purified Tryp‐Psomes were stored for 3 days under three different conditions, at room temperature (rt), 4, and −20 °C (Figure S20, Supporting Information). Afterward, the enzyme activity was checked for each condition at 25 °C and pH 7.5. Tryp‐Psomes A shows a slightly better stability at rt and 4 °C. However, an adequate stability with unchanged enzyme activity at −20 °C is demonstrated for both Tryp‐Psomes even for long‐term storage (4 weeks).

### 2.5 Cryo‐TEM and Asymmetric Flow Field Flow Fractionation Study on Artificial Organelles

**Figure 2:**
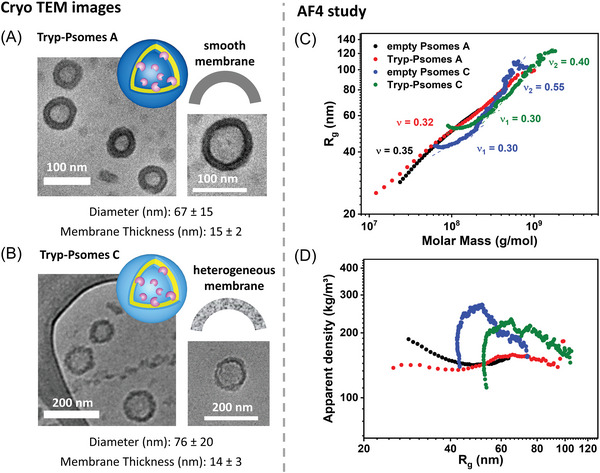
Comparison of structural conformation of both Psomes and their membrane smoothness. A,B) Cryo‐TEM images. Conditions: 0.5 mg mL^−1^ Tryp‐Psomes. C) AF4 measurements of Tryp‐Psomes A and C and Empty‐Psomes after HFF purification in 1 mm PBS: scaling plots, *R*_g_ (circles) versus Molar masses. D) Apparent density is calculated according to molar mass (Mw) and radius (*R*_g_) with the assumption of spherical shape.

Vesicular structures of Tryp‐Psomes were observed using cryo‐TEM under basic conditions (collapsed state, **Figure** **2**A,B). The average diameter and membrane thickness of Tryp‐Psomes A are 67 ± 15 and 15 ± 2 nm, respectively, while the average diameter and membrane thickness of Tryp‐Psomes C are 76 ± 20 and 14 ± 3 nm, respectively. Thus, the diameter of Tryp‐Psomes C is slightly larger than that of Tryp‐Psomes A, but the membrane thicknesses are similar. This indicates that the lumen space of Tryp‐Psomes C might be slightly larger than that of Tryp‐Psomes A.

However, due to the hydrophilic properties of the DMAEMA monomer in Tryp‐Psomes C and permeability at physiological pH (Figure 1D), the membrane of vesicular Tryp‐Psomes C possesses an increased heterogeneity compared to the smooth surface of Tryp‐Psomes A (Figure 2A), showing a partially flower‐like structure (Figure 2B). Asymmetrical flow field flow fractionation in conjunction with light scattering detection (AF4‐LS) was used to better understand the conformational characteristics (e.g., shape and density) of Tryp‐Psomes (Figure 2C,D).^[^ ^35a^ ^]^ Empty‐Psomes A and C were used as a reference.

However, it was considered that the in situ loading process influences the formation of Psomes. First, the radius of gyration, *R*_g_, is plotted versus the molar mass, M, (Figure 2C). The resulting scaling parameter of Psomes A confirms a conformation close to a hard sphere (*ν* = 0.33 for hard sphere). The apparent density is similar for Empty‐Psomes A and Tryp‐Psomes A. Thus, the presence of Tryp during the formation of Psomes A does not affect the structural properties. From Figure S22, Supporting Information, in the early elution range at lower sizes, the *ρ* parameter (*R*_g_/*R*_h_) is ≈1 for Tryp‐Psomes A. Thus, Tryp‐Psomes A can be considered homogenous hollow spheres. When the sizes increase, the value of *ρ* increases as well, which is characteristic of a more heterogeneous conformation caused by aggregation. A *ρ* value of 1 allows to postulate that the enzyme is mainly located in the membrane, giving rise to hollow spheres, and, as seen from the scaling parameter *ν*, the incorporation of the enzyme in the membrane does not disturb the membrane due to its small size or due to partial enzyme location in the Psomes lumen.

In the case of Psomes C, a heterogeneous membrane, observed by cryo‐TEM (Figure 2B), is further corroborated by the scaling parameter *ν* shifting away from the spherical shape in the lower molar mass region to a more heterogeneous conformation for higher molar masses. Nevertheless, after Tryp loading the shape becomes more compact, and the density increases at larger sizes compared to Empty‐Psomes. This observation further supports the preferred membrane‐integration of Tryp in Tryp‐Psomes C. A slightly smaller size for Psomes C is also observed in *R*_g_ value and in the apparent density as found for Tryp‐Psomes A. Furthermore, the consideration of all results (DLS data, including porous state at pH 8; cryo‐TEM and AF4‐LS, shorter self‐assembly time of Psomes formation, and BCP‐C composition with 50 mol% of hydrophilic DMAEMA) for Tryp‐Psomes C allows us to postulate that Tryp‐Psomes C possesses a less compact membrane with pre‐defined porous membrane structure, when explicitly considering the strong differences in the scaling parameter *ν* for perfect vesicles ( = hard spheres with 0.33) and Tryp‐Psomes C as well as the very different vesicular shapes and membranes of Psomes A and C in cryo‐TEM images at pH 8. Overall, the AF4‐LS research highlights a preferential membrane integration of Tryp in both Enzyme‐Psomes.

### 2.6 Macrophage‐ and Lysosome‐like Digestion Functions and Digestion Mechanisms of Artificial Organelles

To study the degradation capacity of biomolecules and the different membrane diffusion in both Tryp‐Psomes, two model enzymes with different sizes, Myo (16.7 kDa) and HRP (44.0 kDa), were chosen and degradation was studied at pH 7.5 and 6.5. Residual enzyme activity was studied using the Amplex Red assay for Myo and ABTS assay for HRP in 1 mm PBS at pH 7.5 and simulated body fluids (SBP (simulated blood plasma) buffer at pH 7.3 and SCF (simulated cerebrospinal fluid) buffer at pH 7.2), respectively (more information in Figure S23, Supporting Information).^[^ ^34^, ^35^ ^]^

**Figure 3:**
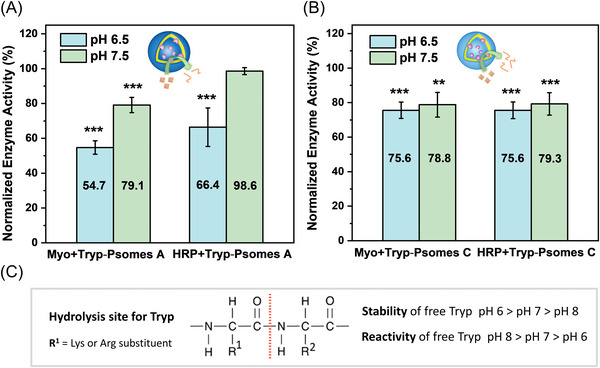
A) Validating lysosome‐ and B) macrophage‐like functions of AOs – The remaining enzyme activity of Myo and HRP incubated with Tryp‐Psomes at pH 6.5 and 7.5 for 24 h. Reference (100%): the remaining enzyme activity of Myo and HRP incubated with Empty‐Psomes at pH 6.5 and 7.5 for 24 h. C) Long‐term stability (≥1 day needed for Tryp‐Psomes use) of free Tryp is even more given between pH 7.5 and 6 and not at pH 8 for an immediate use of freshly prepared Tryp or Tryp‐Psomes solutions. Comparison between both Enzyme‐Psomes is not possible due to different loading efficiency and membrane characteristics. Further details on digesting characteristics for AOs in Figure 4. Significance is indicated with (*) for *p* < 0.05, (**) for *p* < 0.01, and (***) for *p* < 0.001, respectively.

**Figure 4:**
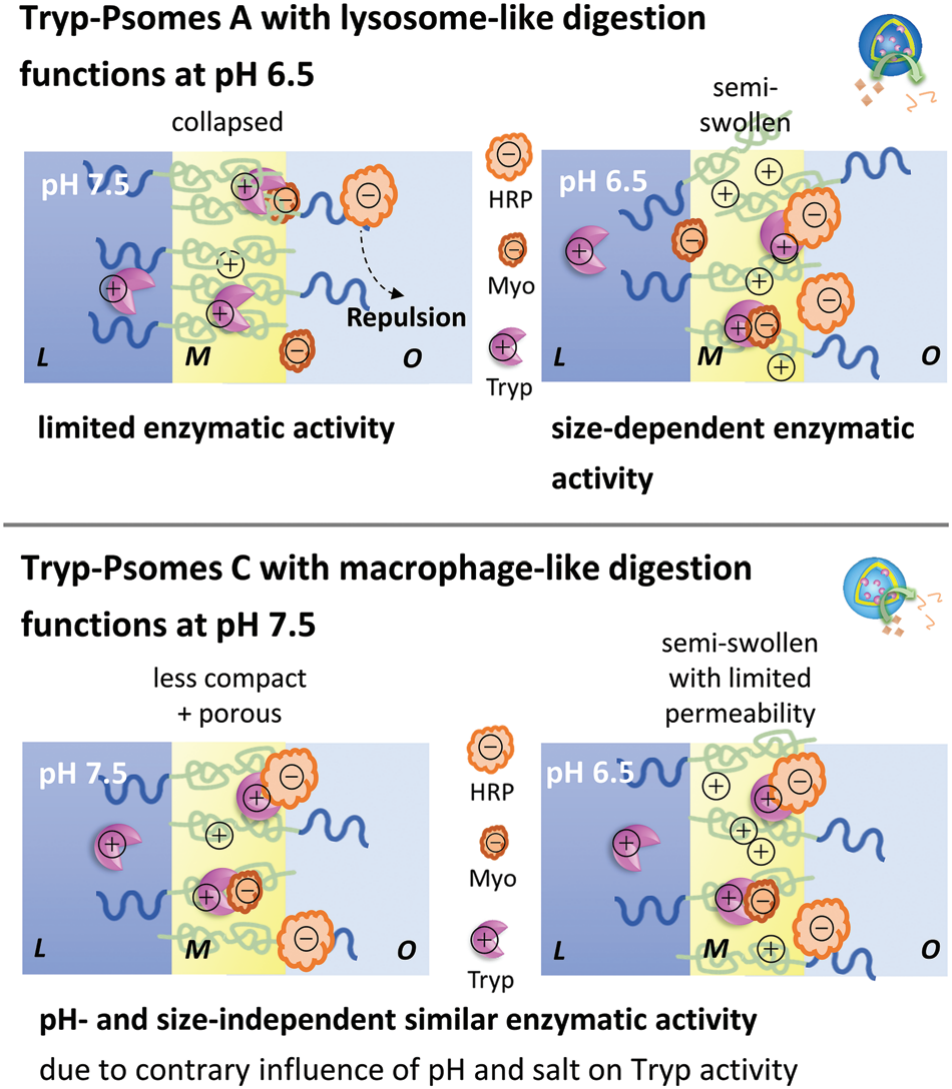
Schematic view on assumed and simplified digestion and membrane characteristics of AOs with lysosome‐ (top) and macrophage‐like (bottom) functions triggered by pH and environmental ion composition; related to the results presented in Figure 3. Possible digestion mechanisms of AOs are discussed at 2.6. and 2.7. L = lumen; M = membrane; O = outside Psomes.

As shown in **Figure** **3**A, Myo (≈45% degradation) and HRP (≈34% degradation) are degraded by Tryp‐Psomes A in the swollen state (pH 6.5; Figure 1D). It is assumed, that partially degraded and free Myo (Table S9, Supporting Information, ∅ ≈10 nm: low degree of Myo aggregation and dynamic disassembly to create single Myo (∅ < 2 nm as like Tryp (Table S4, Supporting Information) during the digestion experiments) are small enough to diffuse into the lumen, allowing its continuous degradation after 24 h of incubation; outlining the same digestion potential as free Tryp (Figure S23, Supporting Information). As previously documented, the size of HRP (Table S9, Supporting Information, ∅ ≈10 nm: low degree of HRP aggregation and dynamic disassembly to create single HRP (∅ < 6 nm) during the digestion experiments) prevents it from diffusing into the lumen.^[^ ^15^, ^32^ ^]^ However, the opened membrane state of Tryp‐Psomes A at pH 6.5 (Figure 1D) allows the physical interaction of HRP with Tryp integrated in the membrane for initiating its degradation at the interface membrane‐PEG shell. This simplified mechanistic view ( = size‐dependent activity) on pathogens degradation of Myo and HRP is briefly visualized in **Figure** **4** (top, right – pH 6.5).

At collapsed membrane state (Figure 1D), Tryp‐Psomes A (Figure 3A – pH 7.5) cannot degrade HRP (≈1%–2% degradation), but some degradation of Myo (≈21%) is possible. The Myo degradation at pH 7.5 is mainly caused by direct physical surface contact of Myo with Tryp at outer the membrane surface of Tryp‐Psomes A due to the small size of single Myo (∅ < 2 nm). We assume that HRP is not degraded because it cannot physically interact with membrane‐integrated Tryp due to repulsion properties of the PEG shell of collapsed Tryp‐Psomes A. A mechanistic model for the limited enzymatic activity for both model pathogens by Tryp‐Psomes A is presented in Figure 4 (top, left – pH 7.5).

Overall, we can conclude that the enzymatic degradation capacity of Tryp‐Psomes A is defined by the pH‐responsive membrane permeability (collapsed versus (slightly) opened (= swollen) state), the biomolecule size, and its direct surface contact with (membrane‐integrated) Tryp. Tryp‐Psomes A outline the desired lysosome‐like digestion and capturing functions at acidic pH, but show no significant digesting functions at pH ≥ 7 (Figure 3A), as postulated in our working concept (Scheme 1). The results outline a type of switching‐on and ‐off digestion characteristics at different pH values and, in particular, a diffusion barrier toward larger biomolecules at both selected pH values and environment (Figure 4, top).

Figure 3B shows that Myo and HRP are degraded by Tryp‐Psomes C at pH 6.5 (both ≈24% degradation) and 7.5 (both ≈21% degradation), and at pH 6.5 the degradation efficiency is a bit higher than at pH 7.5. These results may indicate a slightly lower membrane permeability at pH 7.5 in opposite to semi‐swollen membrane (Figure 1D) with limited permeability at pH 6.5. This explicitly shows the same porous membrane characteristics at each selected pH, outlining a similar capturing potential of pathogens in the membrane scaffold of Tryp‐Psomes C independent on applied pH, as shortly summarized for Tryp‐Psomes C in Figure 4 (bottom). Finally, we can conclude that the membrane‐integrated Tryp in Tryp‐Psomes C, anionic pathogens surface contact (Table S6, Supporting Information) with Tryp by partly crossing membrane from outside to inside or by penetration into (naturally) swollen membrane, and the porous and slightly cationic membrane structure ( = naturally swollen state) of Tryp‐Psomes C (Figure S24, Supporting Information) are the dominant factors for providing the key characteristics of macrophage‐like digesting and capturing functions at physiological pH (Figure 4, bottom).

### 2.7 Additional Aspects of Digestion Mechanisms for Artificial Organelles

Control experiments with pure Tryp in 1 mm PBS at pH 7.5 further confirm the potential of our system as pathogen digesting AOs. Approximately half (≈44%–45% degradation) of the model pathogens, Myo and HRP, are degraded by pure Tryp (Figure S23, Supporting Information) with no differentiation. This implies that the use of Psomes A and C with membrane‐integrated Tryp in combination with the control of membrane permeability is a key requirement for mimicking lysosome‐ or macrophage‐like digestion behavior. Results from zeta potential measurements indicate that a complex charge interplay between cationic membranes of AOs (Figure S24, Supporting Information, cationic surface: Tryp‐Psomes C > Tryp‐Psomes A), (slightly) cationic Tryp (Table S6, Supporting Information) and anionic pathogens (Myo + HRP) (Table S6, Supporting Information) play an important role in the pH sensitive biomimetic cell function (Scheme 1). We postulate that ionic interactions are a driving force to support the formation of surface contacts between Tryp and pathogens for initiating the digestion of model pathogens (Myo + HRP) (Figure 4), once pathogen capturing takes place at and in the membrane. Thus, a limited mobility ( = dynamic location) of Tryp due to membrane interaction between pH 7.5 and 6.5 in both AOs is assumed during the swelling and collapsing events of AOs membrane (Figure 4).

### 2.8 Macrophage‐ and Lysosome‐Like Digestion Functions of Artificial Organelles in Simulated Body Fluids

Thus, it is possible to further modulate the digestion characteristics of both AOs using the extracellular environment (pH and salt composition). In the course of the development of artificial organelles for therapeutic dysfunctional lysosomal applications, it is not always possible to perform in vivo studies (e.g., animal experiments), especially in the early stages that deal with the optimization of the processing, mechanical and structural parameters.

Therefore, enzyme degradation by Tryp‐Psomes A and C in two different simulated body fluids^[^ ^36^ ^]^ was also studied for 24 h (**Table** **2**), using SBP (pH 7.3) and SCF (pH 7.2) to investigate macrophage‐like digestion functions. As pre‐work Empty‐Psomes in the presence of Myo and HRP were used as control to exclude any interferences. The salt compositions of both simulated body fluids (SI 1.13) represent much stronger environmental salt concentrations than the previous experimental series (Figure 3). This may have an additional influence on the macrophage‐like digesting functions of both Tryp‐Psomes. Moreover, additional shielding effects of both model pathogen surfaces can arise resulting in lower contact areas with membrane‐integrated Tryp for inducing the desired protein digesting characteristics at physiological pH (Figure 4, left bottom and left top). As a preliminary control, Myo and HRP degradation using free Tryp was studied in the chosen environments (Figure S23, Supporting Information). Both enzymes can be degraded by pure Tryp, indicating that free Tryp maintains its enzymatic activity in all environments. However, Myo is better protected under the simulated body fluids, showing a lower degradation by Tryp than in 1 mm PBS buffer.

**Table 2:** Validating macrophage‐like functions of Tryp‐Psomes A and C – The remaining enzyme activity of Myo and HRP incubated with Tryp‐Psomes in different simulated body fluids for 24 h. The remaining enzyme activity of Myo and HRP incubated with Empty‐Psomes for 24 h was used as reference (100%)

Incubation with Tryp‐Psomes		Remaining enzyme activity [%]	
		SBP (pH 7.25)	SCF (pH 7.2)
Myo (16.7 kDa)	Tryp‐Psomes A	70.9 ± 14.9	78.3 ± 5.1
	Tryp‐Psomes C	65.8 ±11.2	59.0 ± 15.1
HRP (44 kDa)	Tryp‐Psomes A	83.0 ± 20.1	72.0 ± 20.2
	Tryp‐Psomes C	103.4 ± 7.1	90.8 ± 4.9

**Figure 5:**
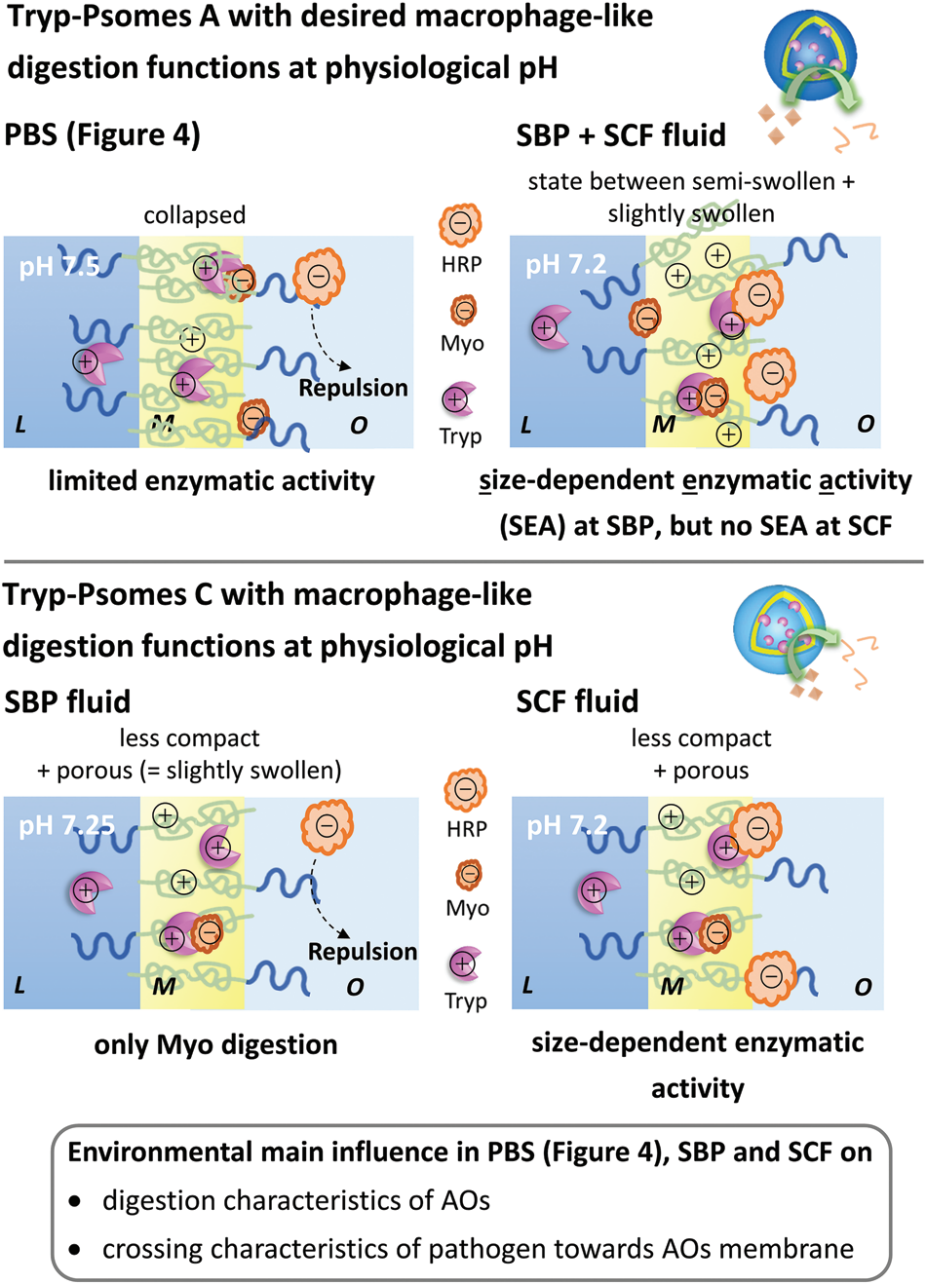
Schematic view on assumed digestion and membrane characteristics of AOs with simplified macrophage‐like digestion functions in simulated body fluids, SBP and SCF triggered by environmental compositions; related to the results presented in Table 2. Possible digestion mechanisms of AOs are discussed at **2.8**. L = lumen; M = membrane; O = outside Psomes.

In the case of SBP fluid (Table 2), the desired digesting characteristics ( = macrophage‐like digestion functions) of both Tryp‐Psomes A and C are much more distinct compared to the experimental series at pH 7.5 (Figure 3). However, HRP is not degraded in the case of Tryp‐Psomes C due to the possible shielding effects of the Tryp‐Psomes C membrane ( = repulsion effect of surface) and/or HRP surface (**Figure** **5**).

In the case of SCF fluid (Table 2), similar digestion characteristics are observed as found in the case of SBP fluid, while Tryp‐Psomes C are able to degrade HRP to a low degree in SCF. Overall, this simulated body fluid experiment series outlines that macrophage‐like digestion function is achieved with both Tryp‐Psomes, however, Tryp‐Psomes C are more selective than Tryp‐Psomes A toward the smaller model pathogen, Myo, in body fluids at physiological pH. We can state similar digestion mechanisms (Figure 5) as suggested in case of PBS (Figure 4), but with variation in model pathogen size dependency of the enzymatic activities (Figure 5). Moreover, we assume that the membrane state of Tryp‐Psomes A can be described between semi‐swollen and slightly swollen due to the strong influence of SCF and SBP ion composition on membrane characteristics of Psomes A (Figure 5), while in case of PBS at pH 7.5 only a collapsed membrane is suggested (Figure 1D). Finally, the interplay of different parameters (e.g., environmental conditions, size of model pathogens, location of Tryp in the membrane, and permeability of Tryp‐Psomes at a specific pH) dictates the appearance of the desired macrophage‐like digestion function of artificial organelles in simulated body fluids (Figure 5). Finally, the results support our goal of achieving macrophage‐like capturing and digesting functions of AOs in different physiological environments.

## 3 Conclusions

In conclusion, we designed and constructed AOs with macrophage‐ and lysosome‐like digestion and capturing functions. It was possible to demonstrate that these AOs are able to digest the model pathogens, Myo and HRP of different sizes, under simulated body fluids, controlled by the environment. To achieve this goal, two cross‐linked and pH‐sensitive Psomes with membrane‐integrated Tryp (Tryp‐Psomes A and C) and with pH‐tunable membrane porosity (membrane composition of pH‐responsive monomer, modulating the hydrophobic/hydrophilic balance) were realized and deeply characterized. Tryp‐Psomes A and C demonstrated to be perfectly stable, storable and equipped with enzyme digestion activity, depending on the pH and salt concentrations. Not only digestion characteristics of AOs are dictated by the environmental conditions, but also the crossing characteristics of pathogens (Myo and HRP) toward the membrane of AOs. It is worth noting that, because of the membrane architecture, Tryp‐Psomes C is active for pathogen digestion at both physiological and acidic pH, a trait not previously observed in pH‐responsive AOs.^[^ ^28^, ^30^, ^34^, ^35^ ^]^ In comparison, Tryp‐Psome A show a clear preferential activity at acidic pH mimicking digestion in lysosomes. This truly demonstrates an increased pH window for potential therapeutically active AOs and paves the way for the use of Tryp‐Psomes as therapeutic artificial organelles with environmental‐dependent lysosome‐ or macrophage‐like capturing and digestion functions making them suited for ERT to overcome the enzymatic lack in many dysfunctional lysosomal diseases.

## Conflict of Interest

The authors declare no conflict of interest.

## Supporting information

Supporting Information

## Data Availability

The data that support the findings of this study are available in the supplementary material of this article.
