# Supplementary material for: Artificial Organelles with Digesting Characteristics: Imitating Simplified Lysosome‐ and Macrophage‐Like Functions by Trypsin‐Loaded Polymersomes
Source: Adv Sci (Weinh). 2023 Apr 19;10(17):2207214. doi: 10.1002/advs.202207214 (PMC10265080; doi:10.1002/advs.202207214)
Supplement: Supplementary file 1 — Supporting Information [file ADVS-10-2207214-s001.pdf]

ADVANCED SCIENCE
Open Access

Supporting Information

for *Adv. Sci.*, DOI 10.1002/advs.202207214

Artificial Organelles with Digesting Characteristics: Imitating Simplified Lysosome- and Macrophage-Like Functions by Trypsin-Loaded Polymersomes

Xiaoying Xu, Silvia Moreno, Susanne Boye, Peng Wang, Brigitte Voit and Dietmar Appelhans**

Supporting Information

Artificial organelles with digesting characteristics: Imitating simplified lysosome- and macrophage-like functions by trypsin-loaded polymersomes

Xiaoying Xu[a, b]*, Silvia Moreno**[a]*, Susanne Boye*[a]*, Peng Wang*[a, b]*, Brigitte Voit*[a, b]*, and Dietmar Appelhans**[a]

[a] Leibniz-Institut für Polymerforschung Dresden e.V., Hohe Straße 6, D-01069 Dresden, Germany

E-Mail: moreno@ipfdd.de, applhans@ipfdd.de

[b] Organic Chemistry of Polymers, Technische Universität Dresden, D-01062 Dresden, Germany

Contents

1. **Experimental Section** **3**

1.1 Materials 3

1.2 Devices 3

1.3 Synthesis and characterization of precursors[3] 6

1.3.1 Synthesis of photo crosslinker (DMIBMA) 6

1.3.2 Synthesis of PEG-Br macroinitiator 7

1.4 Synthesis of BCPs[4] 7

1.5 Fabrication and characterization of pH responsive and crosslinked polymersomes (Empty-Psomes)[3-5] 9

1.6 Control experiments using free Trypsin (Tryp) 10

1.7 Synthesis and characterization of labelled Trypsin 11

1.8 Fabrication and characterization of labelled Tryp-Psomes by *in situ* loading: Loading efficiency and HFF purification process by fluorescence intensity 11

1.9 Fabrication and characterization of Tryp-Psomes: Loading efficiency and stability by enzyme assay 12

1.10 Structural parameters of Tryp-Psomes A by AF4 13

1.11 The degradation of myoglobin (Myo)/horseradish peroxidase (HRP) in the presence of free trypsin 13

1.12 The degradation of Myo/HRP in the presence of Tryp-Psomes 14

1.13 Preparation of simulated blood fluids 16

1.13.1 Preparation of simulated blood plasma[7] 16

1.13.2 Preparation of Artificial cerebrospinal fluid[8] 16

1.14 The degradation of Myo/HRP in the presence of Tryp-Psomes in simulated body fluids 17

2. **Figures and Tables** **18**

3. **Reference** **37**

1. Experimental Section

1.1 Materials

Poly(ethylene glycol) methyl ether (MeO-PEG-OH; M_n = 2000 g mol^{-1}; M_w/M_n = 1.05), 2,2′-bipyridine, 4-aminobutanol, 2-(*N*,*N*′-diethylamino)ethyl methacrylate (DEAEMA), 2-(*N*,*N*′-dimethylamino)ethyl methacrylate (DMAEMA), methacryloylic chloride, 2-bromoisobutyryl bromide, 2-aminoethanol, copper (I) bromide (CuBr), aluminum oxide (neutral, activated), phosphate buffered saline (tablet), sodium hydroxide, dimethyl sulfoxide (DMSO), rhodamine B isothiocyanate (RhB-ITC), myoglobin from equine skeletal muscle (Myo, essentially salt-free, lyophilized powder), peroxidase from horseradish (HRP, essentially salt-free, lyophilized powder), hydrogen peroxide solution (30%) and trypsin from bovine pancreas were purchased from Sigma-Aldrich. 3,4-Dimethylmaleic acid anhydride, toluene, THF, ethyl acetate and chloroform-d were purchased from Acros Organics. *n*-Hexane, hydrochloric acid (37%) and silica gel were purchased from Merck (Germany). Anhydrous 2-butanone (Fluka), triethylamine (Fluka) and anhydrous tetrahydrofuran (THF, Sigma-Aldrich) were stored over a molecular sieve. Cyanin-5 NHS-Ester (Cy5-NHS ester) was purchased from Lumiprobe (Germany). Amplex red was purchased from ThermoFisher. Syringe filters and dialysis membrane were purchased from Carl Roth (Germany). Trypsin Activity Colorimetric Assay Kit was purchased from Sigma-Aldrich. Trypsin Activity Assay Kit (Catalog # 3043) was purchased from Chondrex, Inc.

1.2 Devices

Nuclear Magnetic Resonance (NMR) Spectroscopy. Bruker Advance III 500 spectrometer (Bruker Biospin, Germany) was used for recording ^{1}H NMR (500 MHz) and ^{13}C NMR (126 MHz) spectra using $CDCl_3$ or dimethyl sulfoxide-d_6 (DMSO-d_6) as solvent at room temperature. The chemical shifts were referenced to corresponding solvent signals ($CDCl_3$: δ = 7.26 ppm; DMSO-d_6: δ = 2.50 ppm) and were expressed in ppm.

Gel Permeation Chromatography (GPC). The molar mass distributions (Đ), weight average molecular weight (M_w), number average molecular weight (M_n) of block copolymers were measured using size exclusion chromatography (SEC) equipped with an Agilent 1260 Infinity variable wavelength detector (VWD, Agilent Technologies, Germany) a multi angle laser light scattering (MALLS) detector (DAWN HELEOS II, Wyatt Technology Europe, Germany), a viscometer (Viscostar III, Wyatt Technology Europe, Germany) and a refractive index (RI) detector (Optilab T-rEX, Wyatt Technology Europe, Germany). The column (high-performance

liquid chromatography (HPLC) Column Plgel, Mixed-C, 300 × 7.5 mm, average bead size: 5 μm) and the pump (Isocratic pump, Agilent 1200 series) were from Agilent Technologies (Germany). THF was used as an eluent (stabilized with 0.025 % butylated hydroxytoluene (BHT)) with a flow rate of 1 mL min^{-1}.

Hollow Fiber Filtration (HFF). This filtration technique was used to remove the unbounded or unloaded enzymes during the self-assembly process. HFF was carried out using KrosFlo Research lli System. This device was equipped with a separation module made of polyether sulfone membrane (MWCO: 500 kDa, SpectrumLabs, USA). The transmembrane pressure was 130-110 mbar with a flow rate of 15 mL min^{-1}, while the waste volume is 150-200 mL.

Dynamic Light Scattering (DLS). DLS measurements of aqueous polymersome solutions (≤ 1 mg mL^{-1}) were carried out using a Zetasizer Nano-series instrument (Malvern Instruments, UK) equipped with Dispersion Technology Software (version 5.00). The measurements were carried over a range of pH at 25°C. The data was collected using the NIBS (non-invasive back-scatter) method using a Helium-Neon laser (4 mW, l = 632.8 nm) and a fixed angle of 173°. The data were analyzed using Malvern Software 7.11.

Zeta potential (ζ). ζ measurements were carried out on nanoobjetcs (1 mg mL^{-1} in buffer) at 25°C using a Zetasizer Nano-series instrument (Malvern Instruments, UK) through electrophoretic light scattering. Data evaluation was carried out using Malvern Software 7.11.

Fluorescence intensity. Fluorescence spectra were measured by Fluorolog 3 (Horiba JobinYvon, USA) fluorescence spectrophotometer. RhB: $\lambda_{ex}/\lambda_{em}$ 555/580 nm, Cy5: $\lambda_{ex}/\lambda_{em}$ 646/662 nm.

UV lamp: Crosslinking of polymersomes after the assembling of block copolymers. EXFO OmniCure S2000 (Lumen Dynamics Group Inc., Canada) equipped with a high-pressure mercury lamp as UV source was used for crosslinking.

Asymmetrical Flow Field-Flow Fractionation with Multi Angle Light Scattering (AF4-MALS). AF4-MALS measurements were performed with an Eclipse DUALTEC system (Wyatt Technology Europe, Germany) with 1 mM PBS buffer at pH 7.4 as carrier liquid. The channel spacer made of poly(tetrafluoroethylene) had a thickness of 490 μm, and the channel dimensions were 26.5 cm in length and from 2.1 to 0.6 cm in width. The membranes used as accumulation wall were composed of regenerated cellulose with a molecular weight cut off (MWCO) of 10 kDa (Superon GmbH, Germany). Flow rates were controlled with an Agilent Technologies 1260er series isocratic pump equipped with vacuum degasser. The detection system consists of a MALS detector (DAWN HELEOS II, Wyatt Technology Europe,

Germany) operating at a wavelength of 660 nm with online DLS detector (DynaProNanoStar, Wyatt Technologies, USA) which is an add-on unit connected to the 99° angle of the MALS, a diode array detector (SPD-M20, Shimadzu) and a refractive index (RI) detector (Optilab T-rEX, Wyatt Technology Europe GmbH, Germany) operating at a wavelength of 660 nm. All injections were performed with an autosampler (1260 series, Agilent Technologies Deutschland GmbH). The data collection and calculation of molar masses and radii were performed by Astra 6.1.2.84 software (Wyatt Technologies, USA). The channel flow rate (F_c) was maintained at 0.5 ml min^{-1} for Psome A and 1 mL min^{-1} for Psomes C at 25 °C. The injection volume was set to 150 µL, measurements were performed in triplicate. Psomes A samples were separated by following parameters (separation method A): the separation starts with an isocratic step with a cross flow rate (F_x) of 2.0 mL min^{-1} for 10 min followed by an exponential F_x gradient from 2.0 to 0 mL min^{-1} within 30 min. The last step proceeds without F_x (0 mL min^{-1}) for 5 min. Psomes C samples were separated by following parameters (separation method B): the separation starts with an isocratic step with a cross flow rate (F_x) of 2.5 mL min^{-1} for 5 min followed by an exponential F_x gradient from 2.5 to 0.15 mL min^{-1} within 50 min. The last step proceeds without F_x (0 mL min^{-1}) for 30 min.

Conformation analysis by AF4-MALS

Scaling parameter: By plotting R_g vs M, ν can be determined by the slope of the curve. It gives information about the molecular shape in the used solvent

$$R_g = K \cdot M^{\nu}$$

ν = 0.33 (sphere); ν = 0.5 - 0.6 (random coil macromolecule); ν= 1 (rigid rod)[1]

Apparent density: The interpretation of the apparent density delivers information about molecular density. It is calculated by R_g and M_w (with V as volume fraction, □ as geometrical correction, N_A as Avogadro's number):

$$d_{app,i} = \frac{M_i}{V(R_g)_i \cdot N_A} \cdot \alpha \text{ with } \alpha = \frac{V_{\text{sphere}}(R_g)}{V_{\text{sphere}}(R)} = \frac{R_g^3}{R^3} = \frac{\left(\sqrt{\frac{3}{5}} \cdot R\right)^3}{R^3} = \left(\frac{3}{5}\right)^{\frac{3}{2}}$$

ρ Parameter: The ratio between R_g and R_h delivers valuable information about conformation and shape of molecules, some examples[2]:

ρ = 0.775 (homogeneous and filled sphere);

ρ = 1.78 (random coil, linear chain (good solvent));

ρ = 1.23 (hyperbranched polymer);

$\rho = 2.1$ (Rod (axial ratio = 2.5));

$\rho = 1.0$ (homogeneous hollow capsule).

Cryo-TEM. Cryo-TEM images were acquired using Libra 120 microscope (Carl Zeiss Microscopy GmbH, Oberkochen, Germany) at an acceleration voltage of 120 kV. Samples were prepared by dropping 2-4 µL of polymersomes solution (0.5 or 1 mg mL^{-1} in 1 mM PBS buffer, pH = 7.5 or 8) on copper grids coated with holey carbon foil (so-called Lacey type). The excess of the solution was removed by filter paper; the sample was then rapidly frozen in liquid ethane at -178 °C. The blotting with the filter paper and plunging into liquid ethane were performed in a Leica GP device (Leica Microsystems GmbH, Wetzlar, Germany). All images were recorded in bright field at -172 °C. The diameter and membrane thickness of polymersomes were determined from cryo-TEM images by using Image J software. The average was calculated by analyzing 100 particles for diameter and 20 particles for membrane thickness.

Microplate Reader. Fluorescence intensity and absorbance were measured on a microplate reader (Infinite M Nano by Tecan, Switzerland), and samples were added on a 96-well microplate for the measurement at the desired wavelength range. RhB: $\lambda_{ex}/\lambda_{em}$ 543/580 nm (Measured Emission Wavelength range: 550-650 nm), Cy5: $\lambda_{ex}/\lambda_{em}$ 646/662 nm, Resorufin: $\lambda_{ex}/\lambda_{em}$ 530 ± 12.5 /580 ± 25 nm ($\lambda_{ex}/\lambda_{em}$ 534/586 nm was used in this study). Gain is 100. The absorbance at 405 nm (A405) was measured for both ABTS assay kit for measuring the HRP activity and Trypsin Activity Colorimetric Assay Kit.

Statistical analysis. One-way ANOVA statistical analysis was performed to evaluate the significance of the experimental data. A value of 0.05 was selected as the significance level, and the data were indicated with (*) for $p < 0.05$, (**) for $p < 0.01$, and (***) for $p < 0.001$, respectively.

1.3 Synthesis and characterization of precursors[3]

1.3.1 Synthesis of photo crosslinker (DMIBMA)

Synthesis for step 1: 5.00 g (39.65 mmol) of maleic acid anhydride were dissolved in 120 mL toluene and 3.54 g (39.65 mmol) 4-aminobutanol were added. The mixture was kept at reflux for 2 h at 130 °C in oil bath and the solvent was removed afterwards at reduced pressure. The crude product was purified using flash chromatography with a *n*-hexane/ethyl acetate (50:50 vol-%) mixture giving a white solid (92 % yield) (**Figure S1-S2**).

^{1}H NMR (DMSO-d_6): δ = 4.35 (t, 1H), 3.36 (m, 4H), 1.88 (s, 6H), 1.50 (m, 2H), 1.35 ppm (m, 2H).

^{13}C NMR (DMSO-*d*$_6$): δ = 171.76 (C=O), 136.54 (C), 60.11 (CH_2), 37.17 (CH_2), 29.65 (CH_2), 24.81 (CH_2), 8.36 ppm (CH_3).

Synthesis for step 2: 6.989 g (35.4 mmol) of maleic imide derivative from step 1 were dried in vacuum and then set under an Argon atmosphere. 100 mL of dry THF were added and the flask was cooled with ice. 5.562 g (53.2 mmol) of methacryloyl chloride were dissolved in 3 ml of dry THF before this solution was added to the mixture of maleic derivative. Next, 6.314 g (62.4 mmol) of dry triethylamine were added, the resulting mixture becomes gloomy and the ice was removed. The reaction was carried out for 2 h at 40 °C and aborted by pouring the reaction into water. The water was extracted three times with ethyl acetate. All organic phases were dried over magnesium sulfate, the solids were removed and the solvent was removed at reduced pressure. The crude product was purified using flash chromatography with a *n*-hexane/ethyl acetate (67:33 Vol-%) mixture to give a colorless oil (75 % yield) (**Figure S3-S4**).

^{1}H NMR (DMSO-*d*$_6$): δ = 6.01 (s, 1H), 5.65 (s, 1H), 4.07 (s, 2H), 3.40 (s, 2H), 1.88 (s, 6H), 1.86 (s, 3H), 1.55 ppm (dt, 4H).

^{13}C NMR (DMSO-*d*$_6$): δ = 171.75 (C=O), 166.46 (C=O), 136.59 (C), 135.88 (C), 125.51 (CH_2), 63.65 (CH_2), 36.81 (CH_2), 25.37 (CH_2), 24.61 (CH_2), 17.91 (CH_3), 8.36 ppm (CH_3).

1.3.2 Synthesis of PEG-Br macroinitiator

5.00 g (2.5 mmol) of PEG_{45}-OH were dried in a flask at vacuum and 60 °C for 30 min. The flask was flushed with argon before 50 ml of dry THF were added. 1.43 g (6.25 mmol) of 2-bromoisobutyric acid bromide were dissolved in 3 mL dry THF before this solution was added to the PEG solution. The flask of the resulting reaction solution was now cooled with ice and 0.38 g (3.75 mmol) of dry triethylamine were added. The gloomy mixture was stirred for 40 h at room temperature. The final macroinitiator was precipitated in dry ice-cooled ether and three times recrystallized in ethanol until a white solid was obtained (82 % yield) (**Figure S5**).

^{1}H NMR (DMSO-*d*$_6$): δ = 4.23 (t, 2H), 3.50 (s, 178H), 3.23 (s, 3H), 1.88 ppm (s, 6H).

1.4 Synthesis of BCPs[4]

Synthesis of BCP-A: PEG_{45}-Br (216 mg; 0.1 mmol) and 2,2’-bipyridine (32 mg; 0.2 mmol) were mixed in a Schlenk tube and dried for 5 min in vacuum. DEAEMA (1.33 g; 7.2 mmol) and the corresponding amount of the crosslinker monomer (20 mol-%, 477.6 mg, 1.8 mmol) were dissolved in 3 mL 2-butanone and the solution was added to the PEG_{45}-Br flask afterwards. The mixture was degassed using two freeze-pump-thaw-cycles and flushed with Argon. Then, CuBr (15 mg; 0.1 mmol) was added and the mixture was degassed using three freeze-pump-

thaw-cycles again, backfilled with argon and stirred for 17 h at 50 °C. To abort the polymerization reaction, the mixture was diluted in 3 mL THF and with additional THF filtrated over activated neutral aluminum oxide to remove any copper species. From the resulting gloomy solution, the solvent was transferred to a dialysis membrane (regenerated cellulose, MWCO 5 kDa) and was dialyzed against methanol (technical grade) for three days exchanging the solvent twice a day before it was dried in vacuum to give a sticky polymer (79 % yield).

^{1}H NMR ($CDCl_3$): δ = 4.05 - 3.79 (m, 218H), 3.57 (s, 180H), 3.44 (q, J = 6.4, 4.6 Hz, 46H), 3.31 (s, 3H), 2.69 - 2.42 (m, 533H), 1.81 (d, J = 83.3 Hz, 452H), 1.03 - 0.69 ppm (m, 860H).

Synthesis of BCP-C: the protocol is similar to BCP-A synthesis; however, two different pH responsive monomers were used. The monomer ratio is DEAEMA (667 mg; 3.6 mmol): DMAEMA (566 mg; 3.6 mmol) = 1: 1. PEG_{45}-Br (216 mg; 0.1 mmol) and 2,2'-bipyridine (32 mg; 0.2 mmol) were mixed in a Schlenk tube and dried for 5 min in vacuum. DEAEMA (667 mg; 3.6 mmol) and DMAEMA (566 mg; 3.6 mmol) and the corresponding amount of the crosslinker monomer (20 mol-%, 477.6 mg, 1.8 mmol) were dissolved in 3 mL 2-butanone and the solution was added to the PEG_{45}-Br flask afterwards. The mixture was degassed using two freeze-pump-thaw-cycles and flushed with Argon. Then, CuBr (15 mg; 0.1 mmol) was added and the mixture was degassed using three freeze-pump-thaw-cycles again, backfilled with argon and stirred for 17 h at 50 °C. To abort the polymerization reaction, the mixture was diluted in 3 mL THF and with additional THF filtrated over activated neutral aluminum oxide to remove any copper species. From the resulting gloomy solution, the solvent was transferred to a dialysis membrane (regenerated cellulose, MWCO 5 kDa) and was dialyzed against methanol (technical grade) for three days exchanging the solvent twice a day before it was dried in vacuum to give a sticky polymer (75 % yield).

^{1}H NMR ($CDCl_3$): δ = 4.20 - 3.85 (m, 227H), 3.66 (s, 180H), 3.55 (d, J = 8.6 Hz, 48H), 3.40 (s, 3H), 2.84 - 2.51 (m, 366H), 2.39 - 2.23 (m, 271H), 1.15 - 0.77 ppm (m, 609H).

The composition and the number average molecular weight (M_n) of the block copolymers were determined with ^{1}H NMR spectroscopy from the peak integrals. Additionally, the molar mass distributions (Đ) were determined by SEC as described in previous section. **Table S1** and **Figure S6-S8** show the corresponding results.

1.5 Fabrication and characterization of pH responsive and crosslinked polymersomes (Empty-Psomes)[3-5]

Empty-Psomes A and Empty-Psomes C formation. The block copolymer (BCP-A or BCP-C; 1 mg BCP mL^{-1}) was dissolved in hydrochloric acid (0.01 M) at pH 2. The solution was passed through a syringe filter (Nylon, 0.2 μm) to remove any remaining particles, including dust. Then following titrated with sodium hydroxide solution (1 M or 0.1 M) (also passed through a syringe filter (Nylon, 0.2 μm)) until pH 8-9 was reached. The solution becomes gloomy in presence of polymeric vesicles. The mixture was stirred in the dark for 1-3 days (the self-assembling process depends on the sample) and passed through a syringe filter (cellulose ester, 0.8 μm).

Crosslinking of Empty-Psomes. The prepared Empty-Psomes solution was placed in the UV chamber, following the 3 min irradiation due to old UV lamp (**Table S2**; for first experiment series), each time 2 mL at a concentration of 1 BCP mg mL^{-1} were used. With the exchange of new UV lamp having higher starting power only an irradiation time up to 60 is needed. Overall the crosslinking time is controlled by an iterative step for checking the power of UV-lamp every two months in our laboratory; without losing the power and characteristics of AOs in the different experiment series. Moreover, crosslinking time is always optimized for newly synthesized block copolymer first and then adapted to the loss of UV lamp power over time. Data for Empty-Psomes in **Table 1** are obtained by crosslinking with 180 s, while all other results (e.g. Enzyme-Psomes) were obtained by $\leq$ 60 s.

Reversible swelling of Empty-Psomes upon repeated changes in pH. To Empty-Psomes solutions, little amounts of 1 M HCl or 1 M NaOH were added to reach pH 5 or 8, respectively. This cycle was repeated 5 times (**Figure S9**). *Conditions:* Empty-Psomes solution (1 mg BCP mL^{-1}, 3 min crosslinking) in 10 mM NaCl.

Determination of pH* (= half power of Psomes swelling). A stock solution of Empty-Psomes was titrated automatically connected to a DLS measurement cell where data were collected after the pH value remained constantly. *Conditions:* Empty-Psomes solution (1 mg BCP mL^{-1}, 3 min crosslinking) in 10 mM NaCl, 1 mM PBS (without NaCl or in presence of 13.7 mM NaCl) or 10 mM PBS (without NaCl or in presence of 137 mM NaCl). DLS titration curves of the assembled vesicles are shown accompanied by the determination of the critical pH value (pH*) by fitting of the DLS data (**Figure S10-11, Figure S24**).

1.6 Control experiments using free Trypsin (Tryp)

a) Trypsin Activity Colorimetric Assay Kit

The technical bulletin of the kit from Sigma-Aldrich is referred in the enzyme experiment. Trypsin is a member of the serine protease family. It cleaves proteins and peptides into smaller pieces by hydrolyzing peptide bonds at the carboxyl side of lysine and arginine residues. Trypsin is produced by the pancreas as an inactive trypsinogen and is then secreted into the small intestine, where it is cleaved by enteropeptidase and becomes activated. Trypsin activity aberration is implicated in gastrointestinal disorders such as pancreatitis and intestinal mucosal pathology.

Trypsin Activity Colorimetric Assay Kit offers a rapid and sensitive way to determine the trypsin activity in mammalian cell/tissue lysates, serum, plasma and other biological fluid samples. Briefly, trypsin cleaves the substrate and releases *p*-nitroanilide (pNA), a chromophore that can be measured at 405 nm using a spectrophotometer.

b) Free trypsin activity

A Tryp stock solution (0.2 mg mL^{-1}) in Milli-Q water was prepared and incubated for 1-2 h, afterwards it was filtrated using 0.2 μm filter. Then, a series of Tryp solutions was prepared using the buffer solution provided by the kit: 50, 100, 200, 300 and 400 ng mL^{-1}. First, 50 μL of different Tryp solutions were added in the wells, next 50 μL of substrate solution were added (2 μL substrate + 48 μL buffer). Total volume in each well was 100 μL. After mixing well, the absorbance was studied at 405 nm at 25 °C for 1.5 h using the microplate reader. The results are shown at the **Figure S12**.

c) pH influence on the enzyme activity and stability

A Tryp stock solution (0.2 mg mL^{-1}) in Milli-Q water was prepared and incubated for 1-2 h, afterwards it was filtrated using 0.2 μm filter. Then Tryp solution was diluted at different pH (6, 7 and 8) in 1 mM PBS buffer (300 ng mL^{-1}) and incubated for 1 hour or 1 day. Afterwards, the Tryp activity was studied directly (**Figure S13**).

d) Stability study using enzyme assay and DLS

Trypsin was dissolved in 1 mM PBS buffer at pH 7.4 (1 mg mL^{-1}, filtrate using 0.2 μm filter) and incubated in the room temperature by slightly stirring. The hydrodynamic diameter of free trypsin was studied by DLS after 1, 2 and 3 days (**Table S4**). Its residual enzyme activity was studied after 1, 3 and 7 days (**Figure S14**).

1.7 Synthesis and characterization of labelled Trypsin

Synthesis of RhB-Trypsin (RhB-Tryp). 30 μL of RhB-ITC solution (0.60 mg, 1.26 mmol, 3 eq, stock = 2 mg in 100 μL DMSO) were added into Tryp solution (0.420 mmol, eq. 1, 10 mg dissolved in 1 mL carbonate buffer solution, pH=8.5). The solution was stirred for 48 h in the dark and then dialyzed using 2 kDa MWCU membrane for 1 day against 1 mM PBS and 1 day against water. Finally, the solution was filtered using 0.8 μm filter and stored at 4 °C.

Synthesis of Cy5-Trypsin (Cy5-Tryp). 100 μL of Cy5-NHS ester solution (0.7 mg, 1.26 mmol, 3 eq, stock = 4.5 mg in 300 μL DMSO) were added into Tryp solution (0.420 mmol, eq. 1, 10 mg dissolve in 1 mL carbonate buffer solution, pH=8.5). The solution was stirred for 48 h in the dark and then dialyzed using 2 kDa MWCU membrane for 1 day against 1 mM PBS and 1 day against water, Finally, the solution was filtered using 0.8 μm filter and stored at 4 °C to obtain Cy5-labelled trypsin solution.

According to the calibration curves by UV-VIS spectroscopy, every trypsin has been labelled 1.6 RhB for RhB-Tryp, every trypsin has been labelled 0.8 Cy5 for Cy5-Tryp. (**Figure S17**).

1.8 Fabrication and characterization of labelled Tryp-Psomes by *in situ* loading: Loading efficiency and HFF purification process by fluorescence intensity

Fabrication. [5-6] 9 mg of BCP were dissolved in 8 mL of HCl 0.01 M (pH 2, 1.12 mg mL^{-1}). The solution was passed through a 0.2 μm nylon filter to remove all impurities. Next, to 7.2 mL of this solution the pH was increased from 2 to 5 by the addition of NaOH 1 M. Then, 800 μL of enzyme (2 mg mL^{-1}, RhB-Tryp, filtrate using 0.2 μm filter) were added obtaining a solution of 1 mg BCP mL^{-1} and 0.2 mg mL^{-1} of enzyme (V_F = 8 mL). Next, pH was increased to 8.5 with NaOH 0.1 M. The suspension was left stirring in the dark for 24 h. Afterwards, the solution was passed through a 0.8 μm nylon filter and crosslinked for 180 s (per 2 mL of suspension). After crosslinking, the size and polydispersity of Tryp-Psomes were checked by batch DLS.

Purification by HFF. The device for HFF purification was equipped with a separation module made of polyether sulfone membrane (MWCO: 500 kDa, SpectrumLabs, USA). The transmembrane pressure was 110 mbar with a flow rate of 15 mL min^{-1}, the buffer was 1 mM PBS, pH 7.5, the waste volume was 150-200 mL until the free dye was totally removed.

Release study using purified dye-labelled Tryp-Psomes. To check the stability and Tryp retention in purified dye-labelled Tryp-Psomes, the release behaviour was studied by dialysis

(1000 kDa membrane) using purified labelled Tryp-Psomes by HFF. The sample (1 mg mL^{-1}) was dialyzed in 1 mM PBS buffer at pH = 7.5 for 24 h, then 1 mL of sample was taken. Later, the sample was dialyzed in 1 mM PBS buffer at pH = 7 for 24 h, then 1 mL of sample is taken. Finally, the sample was dialyzed in 1 mM PBS buffer at pH = 6 for 24 h, then 1 mL of sample is taken. After each process, the residual loading efficiency was studied by fluorescence intensity by triplicate (**Figure S18**). The parameter for measurement: RhB: $\lambda_{ex}/\lambda_{em}$ 543/580, Gain: 80-100, Excitation Bandwidth: 9 nm, Emission Bandwidth: 20 nm.

1.9 Fabrication and characterization of Tryp-Psomes: Loading efficiency and stability by enzyme assay

Fabrication[5]. 9 mg of BCP were dissolved in 8 mL of HCl 0.01 M (pH 2, 1.12 mg mL^{-1}). The solution was passed through a 0.2 µm nylon filter to remove all impurities. Next, to 7.2 mL of this solution the pH was increased from 2 to 5 by the addition of NaOH 1 M. Then, 800 µL of enzyme (2 mg mL^{-1} dissolved in 1 mM PBS buffer, pH = 7.4, filtrate using 0.2 µm filter, trypsin incubation for 1-2 h) were added obtaining a solution of 1 mg mL^{-1} and 0.2 mg mL^{-1} of enzyme (V_F = 8 mL). Next, pH was increased to 9 with NaOH 0.1 M. The suspension was left stirring in the dark for 24 h. Afterwards, the solution was passed through a 0.8 µm nylon filter and crosslinked for 180 s (per 2 mL of suspension). After crosslinking, the size and polydispersity of the Tryp-Psomes were checked by batch DLS. To check the enzyme activity, Tryp-Psomes solution was diluted 15 or 20 times to get the reasonable range of values.

Enzyme activity: Stability. The enzyme activity of purified Tryp-Psomes (fresh sample) was studied in comparison with three samples storage at room temperature, at 4 °C and at -20 °C for 3 days. After that, additional storages times were investigated: a) Short term storage: at 4 °C for 1, 3 and 5 days, B) Longer term storage: at -20 °C for 1, 2, 3 and 4 weeks (**Figure S20**). The study was carried out at pH 7.5 by triplicate.

Enzyme activity: pH dependence. The enzyme activity of purified Tryp-Psomes (fresh sample) was studied at pH 8, 7 and 6 in 1 mM PBS buffer (**Figure S15**). The enzyme activity of purified Tryp-Psomes (fresh sample) was studied at pH 7.5 and 6.5 in 1 mM PBS buffer (**Figure S16**). The study was carried out by duplicate.

Enzyme activity: loading efficiency. The enzyme activity of Tryp-Psomes at pH 7.5 before and after HFF purification was studied. In order to study the loading efficiency after HFF, a calibration curve was carried out using the unpurified sample: Dilute Tryp-Psomes (0.5 mg mL^{-1} Psomes, 0.1 mg mL^{-1} Tryp) without HFF purification 10 times as original solution, and prepare

a series of concentrations sample which included 2, 4, 6, 8 and 10 μg mL^{-1} Tryp. Finally, the enzyme activity of the mentioned samples and HFF purified Tryp-Psomes (0.5 mg mL^{-1} Psomes, 0.1 mg mL^{-1} Tryp) were studied. Use the △A405 value of mentioned series solution to do the calibration curve. The loading efficiency of the Tryp-Psomes A and Tryp-Psomes C after HFF purification is 9.32 % and 9.02 % calculating from the calibration curve (**Figure S19**). The study was carried out by duplicate.

Enzyme activity: Comparing free Tryp versus Tryp-Psomes C. After self-assembly process of Tryp-Psomes C, take 2 mL solution of unpurified Tryp-Psomes C (1 mg mL^{-1}) for crosslinking 60 s (UV lamp with using time of about 33 h). Then take 2 mL solution of free Tryp (0.2 mg mL^{-1} in 1 mM PBS at pH 7.5) for crosslinking 60 s (UV lamp with using time of about 33 h). Using Trypsin Activity Colorimetric Assay Kit buffer to dilute free Tryp, free Tryp after crosslinking (Tryp-CL), Tryp-Psomes C and Tryp-Psomes C after crosslinking (Tryp-Psomes C-CL) to 2 μg mL^{-1} and 0.2 μg mL^{-1}, respectively. Then checking the trypsin activity of all samples via absorbance measurement at λ 405 nm on the microplate reader.

1.10 Structural parameters of Tryp-Psomes A by AF4

The following samples were studied by AF4 (**Figure S21-22**): (a) Non-purified Empty-Psomes A (0.5 mg BCP mL^{-1}); (b) HFF-purified Empty-Psomes A (0.5 mg BCP mL^{-1}); (c) Non-purified *in situ* Tryp-Psomes A (0.5 mg BCP mL^{-1} + 0.1 mg mL^{-1} of Tryp); (d) Purified *in situ* Tryp-Psomes A (0.5 mg BCP mL^{-1} + ≥ 0.1 mg mL^{-1} of Tryp); (e) Non-purified Empty-Psomes C (0.5 mg BCP mL^{-1}); (f) HFF-purified Empty-Psomes C (0.5 mg BCP mL^{-1}); (g) Non-purified *in situ* Tryp-Psomes C (0.5 mg BCP mL^{-1} + 0.1 mg mL^{-1} of Tryp); (h) Purified *in situ* Tryp-Psomes C (0.5 mg BCP mL^{-1} + ≥ 0.1 mg mL^{-1} of Tryp). Control of free enzyme: (a) Free Tryp (0.1 mg mL^{-1}); (b) Free Tryp filtered by 0.2 μm filter (0.1 mg mL^{-1}). All samples were studied at 1 mM PBS at pH 7.4 in presence of 200 mg L^{-1} of NaN_3.

1.11 The degradation of myoglobin (Myo)/horseradish peroxidase (HRP) in the presence of free trypsin

Degradation of Myo

All stock solutions were stored at 4 °C for not more than two weeks.

Stock solutions: C_{Myo}= 2 mg mL^{-1} in 1 mM PBS; C_{Tryp}= 2 mg mL^{-1} in 1 mM PBS; C_{H2O2} = 1 M in Milli-Q water; C_{H2O2} = 0.02 M in Milli-Q water; Amplex red (dilute the stock solution (DMSO) to 0.02 mg mL^{-1} in Milli-Q water).

Buffer (degradation and enzyme activity): 1 mM PBS at pH 7.5; simulated blood plasma (SBP) buffer, pH 7.3; simulated cerebrospinal fluid (SCF) buffer, pH 7.2.

The buffer was always filtrated using 0.2 μm filter before each usage.

(1) **Control:** 0.02 mg mL^{-1} Myo (20 μL Myo + 1.98 mL buffer);

(2) 0.02 mg mL^{-1} Myo + 0.04 mg mL^{-1} Tryp (20 μL Myo + 40 μL Tryp + 1.94 mL buffer).

The samples were incubated in the dark for 24 h.

Residual enzyme activity of Myo. An aliquot of 300 μL of the prepared solutions was treated with 3 μL of Amplex red (0.02 mg mL^{-1} in Milli-Q water) and 3 μL of H_2O_2 (0.02 M in Millipore). The mixture was vigorously shaken and after 15 min, the fluorescence spectra were recorded at an excitation wavelength of 534 nm was recorded immediately. Each assay was executed in triplicates (**Figure S23**).

Degradation of HRP

All stock solutions were stored at 4 °C for not more than two weeks.

Stock solutions: C_{HRP}= 2 mg mL^{-1} in 1 mM PBS; C_{Tryp}= 2 mg mL^{-1} in 1 mM PBS; C_{H2O2} = 1 M in Milli-Q water; C_{H2O2} = 0.02 M in Milli-Q water; C_{ABTS} = 0.02 M in Milli-Q water.

Buffer (degradation and enzyme activity): 1 mM PBS at pH 7.5; simulated blood plasma (SBP) buffer, pH 7.3; simulated cerebrospinal fluid (SCF) buffer, pH 7.2.

The buffer was always filtrated using 0.2 μm filter before each usage.

(1) **Control:** 0.02 mg mL^{-1} HRP (20 μL HRP + 1.98 mL buffer);

(2) 0.02 mg mL^{-1} HRP + 0.04 mg mL^{-1} Tryp (20 μL HRP + 40 μL Tryp + 1.94 mL buffer).

The samples were incubated in the dark for 24 h.

Residual enzyme activity of HRP. An aliquot of 300 μL of the prepared solutions was treated with 3 μL of H_2O_2 (0.02 M in Millipore) and 3 μL of ABTS (0.02 M in Millipore). The mixture was vigorously shaken and after 30 min the UV absorbance at 405 nm was recorded immediately, each assay is executed in triplicates (**Figure S23**).

1.12 The degradation of Myo/HRP in the presence of Tryp-Psomes

Degradation of Myo by purified Tryp-Psomes

Stock solutions: C_{Stock_Myo}= 2 mg mL^{-1} in 1 mM PBS; $C_{Stock\_Tryp\text{-}Psomes}$ = 1 mg mL^{-1} in 1 mM PBS; $C_{Stock\_Empty\text{-}Psomes}$ = 1 mg mL^{-1} in 1 mM PBS.

Sample 1-2 (Control): Empty-Psomes (0.5 mg mL^{-1}) + Myo (0.02 mg mL^{-1})

20 μL of Myo solution (2 mg mL^{-1} dissolve in water) and 980 μL of 1 mM PBS buffer (pH 7.5) were added to 1 mL HFF purified Empty-Psomes (1 mg mL^{-1}) solution. The solution was split into two samples, then adjust the mixture solution pH, one to 6.5, another one to 7.5.

Sample 3-4: Tryp-Psomes (0.5 mg mL^{-1}) + Myo (0.02 mg mL^{-1})

20 μL of Myo solution (2 mg mL^{-1} dissolve in water) and 980 μL of 1 mM PBS buffer (pH 7.5) were added to 1 mL HFF purified Tryp-Psomes (1 mg mL^{-1}) solution. The solution was split into two samples, then adjust the mixture solution pH, one to 6.5, another one to 7.5.

All samples were incubated in the dark for 24 h. After 24 h, 300 μL of sample were taken into the Eppendorf tube then 300 μL of 1 mM PBS buffer (pH = 6.5 or 7.5), 3 μL of Amplex red (0.01 mg mL^{-1} in Millipore) and 3 μL of H_2O_2 (0.01 M in Millipore) were added. The samples were incubated in the dark for 15 min. Finally, 600 μL of 10 mM PBS buffer (pH 7.5) were added into the tube to adjust all the samples pH to 7.5. The fluorescence spectra were recorded at an excitation wavelength of 534 nm immediately, each assay was executed in triplicates **(Figure 3**).

Degradation of HRP by purified Tryp-Psomes

Stock solutions: C_{Stock_HRP}= 2 mg mL^{-1} in 1 mM PBS; $C_{Stock\_Tryp\text{-}Psomes}$ = 1 mg mL^{-1} in 1 mM PBS; $C_{Stock\_Empty\text{-}Psomes}$ = 1 mg mL^{-1} in 1 mM PBS.

Sample 1-2 (control): Empty-Psomes (0.5 mg mL^{-1}) + HRP (0.02 mg mL^{-1})

20 μL of HRP solution (2 mg mL^{-1} dissolve in water) and 980 μL of 1 mM PBS buffer (pH 7.5) were added to 1 mL HFF purified Empty-Psomes (1 mg mL^{-1}) solution. The solution was split into two samples, then adjust the mixture solution pH, one to 6.5, another one to 7.5.

Sample 3-4: Tryp-Psomes (0.5 mg mL^{-1}) + HRP (0.02 mg mL^{-1})

20 μL of HRP solution (2 mg mL^{-1} dissolve in water) and 980 μL of 1 mM PBS buffer (pH 7.5) were added to 1 mL HFF purified Tryp-Psomes (1 mg mL^{-1}) solution. The solution was split into two samples, then adjust the mixture solution pH, one to 6.5, another one to 7.5.

All samples were incubated in the dark for 24 h. After 24 h, 300 μL of sample were taken into the Eppendorf tube then 300 μL of 1 mM PBS buffer (pH = 6.5 or 7.5), 3 μL ABTS (0.01 M in Millipore) and 3 μL of H_2O_2 (0.01 M in Millipore) were added. The samples were incubated in the dark for 15 min. Finally, 600 μL of 10 mM PBS buffer (pH 7.5) were added into the tube

to adjust all the samples pH to 7.5. After preparation, the UV monitoring at 405 nm was started and data points were recorded immediately, each assay is executed in triplicates **(Figure 3**).

1.13 Preparation of simulated blood fluids

1.13.1 Preparation of simulated blood plasma[7]

50 mL of simulated blood plasma were prepared, at pH = 7.3 (adjust it if needed). The buffer was always filtrated with 0.2 µm PA filter before experiments:

Chemical	Mass [mg]
NaCl	401.75
$NaHCO_3$	17.75
KCl	11.25
K_2HPO_4	11.55
$MgCl_2$	15.55
1 M HCL	1.95
$CaCl_2$	14.6
Na_2SO_4	3.6
TRIS	305.9

1.13.2 Preparation of Artificial cerebrospinal fluid[8]

Artificial cerebrospinal fluid (ACSF) is commonly used as a vehicle solution for administration of test agents to the central nervous system (CNS) of laboratory animals. Listed below is a suggested method for the preparation of artificial cerebrospinal fluid. This solution closely matches the electrolyte concentrations and physiological compatibility of endogenous CSF. (Reference from: https://www.alzet.com/guide-to-use/preparation-of-artificial-csf/). Remark: In this paper, we name artificial cerebrospinal fluid as simulated cerebrospinal fluid buffer (SCF buffer).

Preparation of Solution A: weigh appropriate amounts of each compound listed below and **dissolve in 500 mL** pyrogen-free, sterile water:

Chemical	**Mass** (g)
NaCl	8.66
KCl	0.224
$CaCl_2 \cdot 2H_2O$	0.206
$MgCl_2 \cdot 6H_2O$	0.163

Preparation of Solution B: weigh appropriate amounts of each compound listed below and **dissolve in 500 ml** pyrogen-free, sterile water:

**Chemical**	**Mass** (g)
$Na_2HPO_4 \cdot 7H_2O$	0.214
$NaH_2PO_4 \cdot H_2O$	0.027

Preparation of Artificial cerebrospinal fluid (simulated cerebrospinal fluid buffer, SCF buffer): combine solutions A and B in a 1:1 ratio, pH = 7.2. Equal volumes of each solution are required to end up with a multivalent physiological ion solution of a SCF buffer.

1.14 The degradation of Myo/HRP in the presence of Tryp-Psomes in simulated body fluids

Stock solutions: $C_{Stock\_enzyme(Myo\ or\ HRP)}$= 2 mg mL^{-1} in 1 mM PBS; $C_{Stock\_Try\text{-}Psomes\ =}$ 1 mg mL^{-1} in 1 mM PBS; $C_{Stock\_Empty\text{-}Psomes\ =}$ 1 mg mL^{-1} in 1 mM PBS.

Sample 1-2: Empty-Psomes (0.5 mg mL^{-1}) + HRP or Myo (0.02 mg mL^{-1})

20 µL of HRP or Myo solution (2 mg mL^{-1} dissolve in 1 mM PBS) and 980 µL of simulated buffer were added to 1 mL HFF purified Empty-Psomes (1 mg mL^{-1}) solution.

Sample 3-4: Tryp-Psomes (0.5 mg mL^{-1}) + HRP or Myo (0.02 mg mL^{-1})

20 µL of HRP or Myo solutions (2 mg mL^{-1} dissolve in water) and 980 µL of simulated buffer were added to 1 mL HFF purified Tryp-Psomes (1 mg mL^{-1}) solution.

The incubation and measurement were the same as the mentioned above **(Table 2**).

2. Figures and Tables

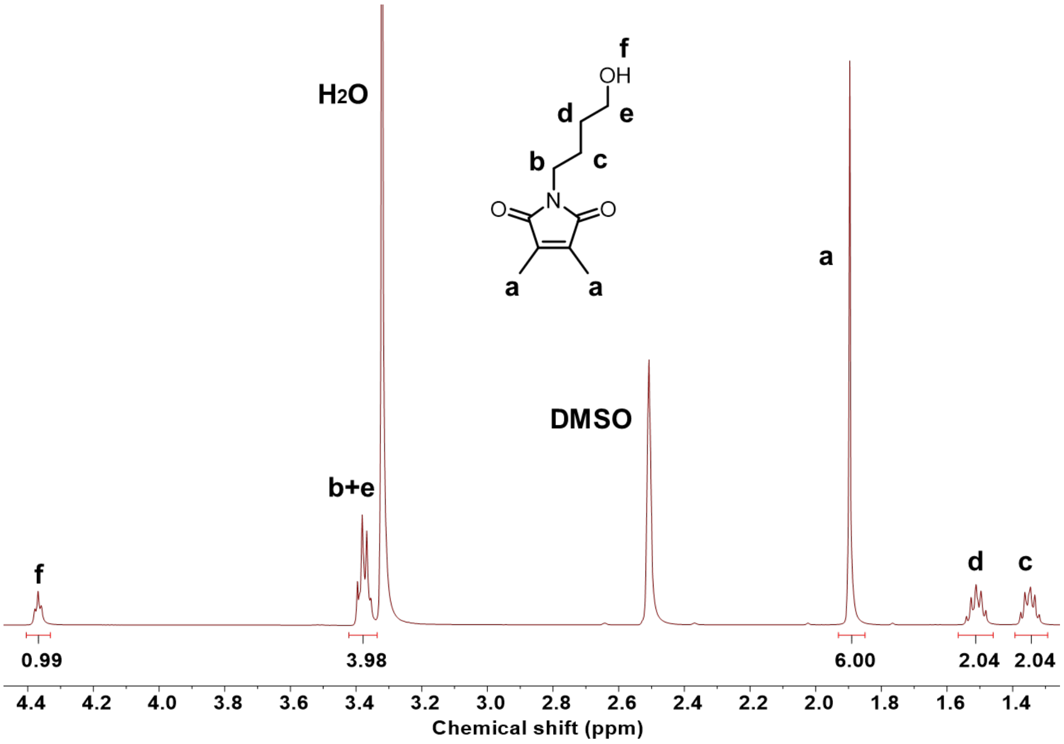

Figure S1. ^{1}H NMR spectrum of Crosslinker DMIBMA (Step 1) in DMSO-d6.

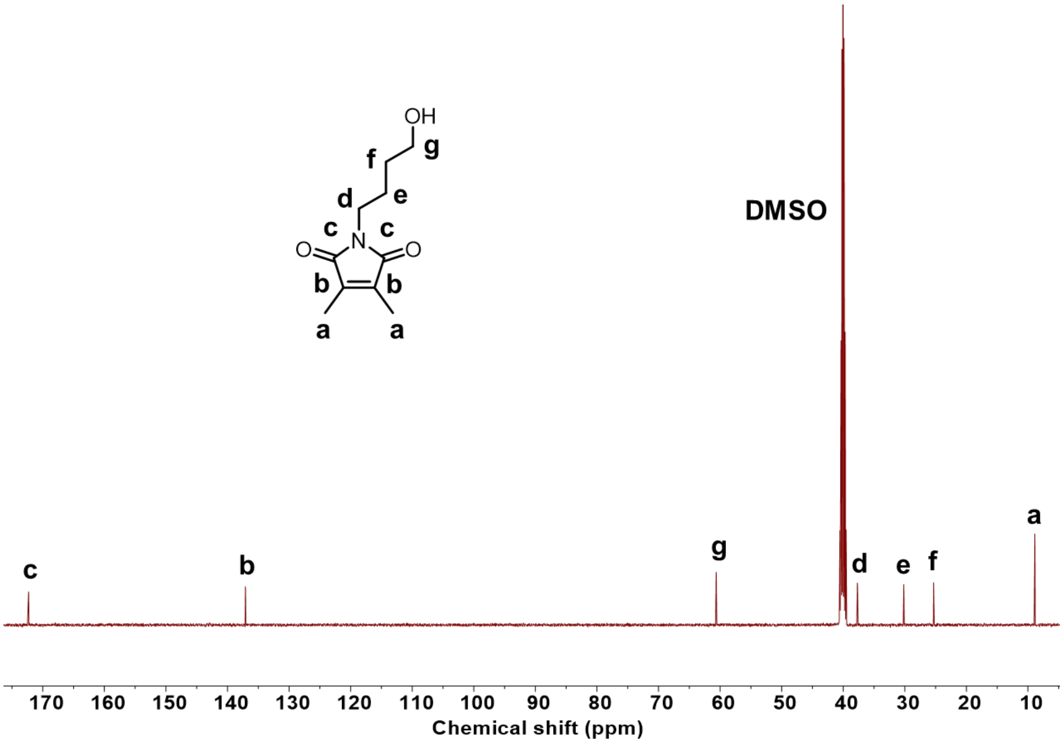

Figure S2. ^{13}C NMR spectrum of Crosslinker DMIBMA (Step 1) in DMSO-d6.

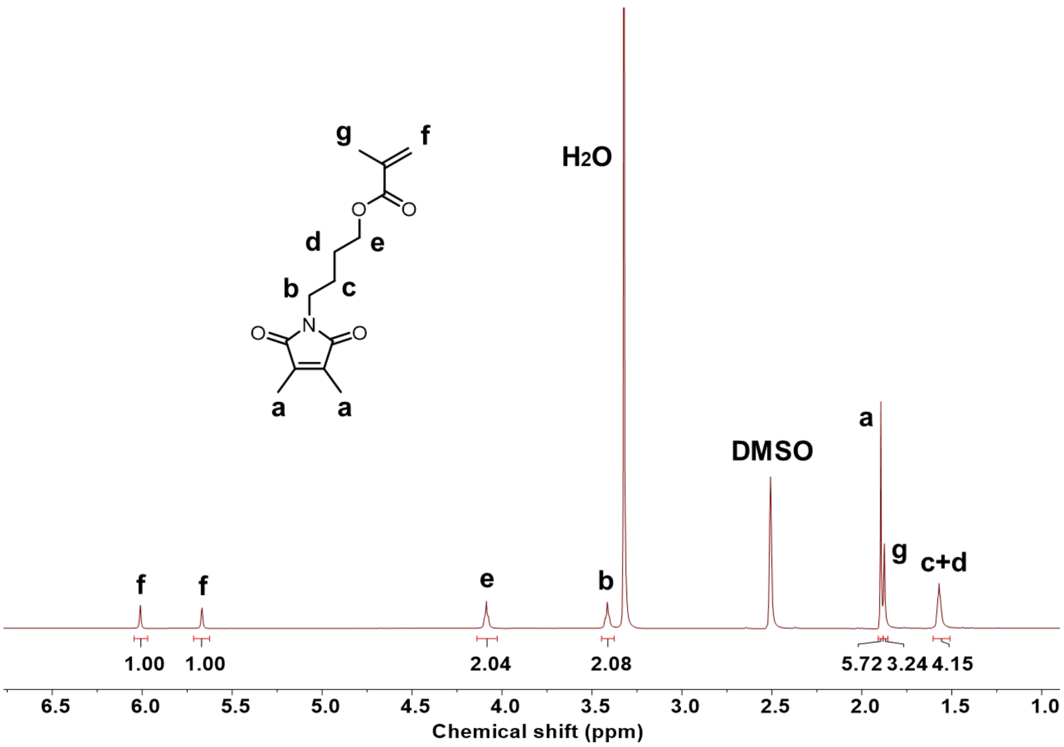

Figure S3. ^{1}H NMR spectrum of Crosslinker DMIBMA (Step 2) in DMSO-d_6.

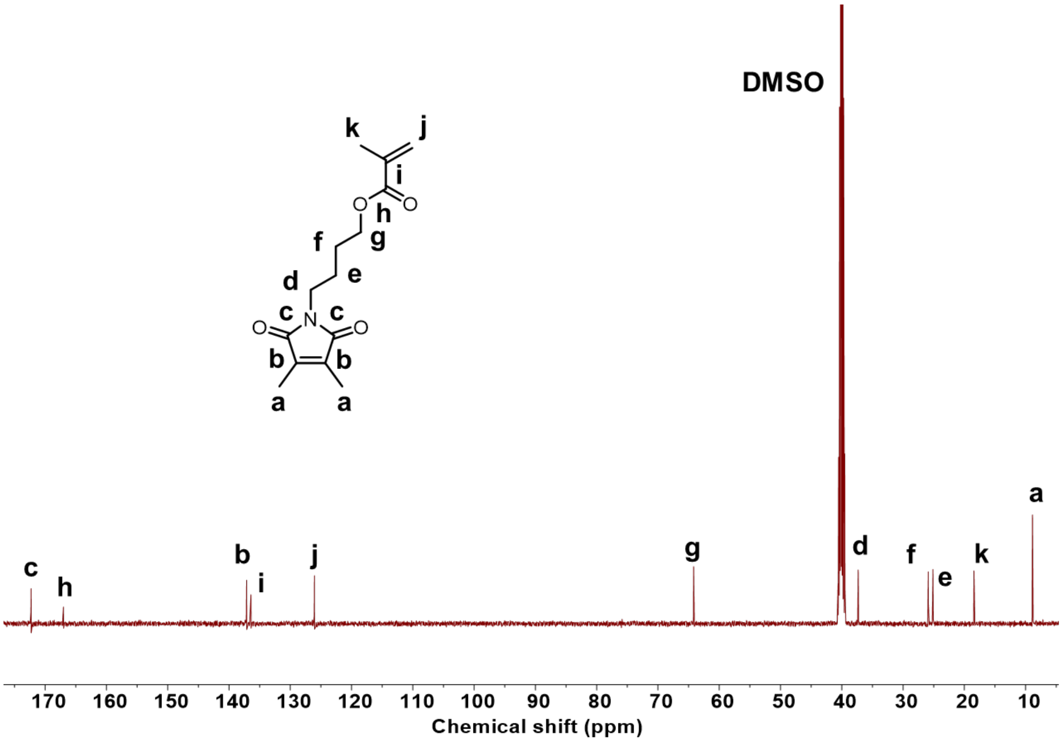

Figure S4. ^{13}C NMR spectrum of Crosslinker DMIBMA (Step 2) in DMSO-d_6.

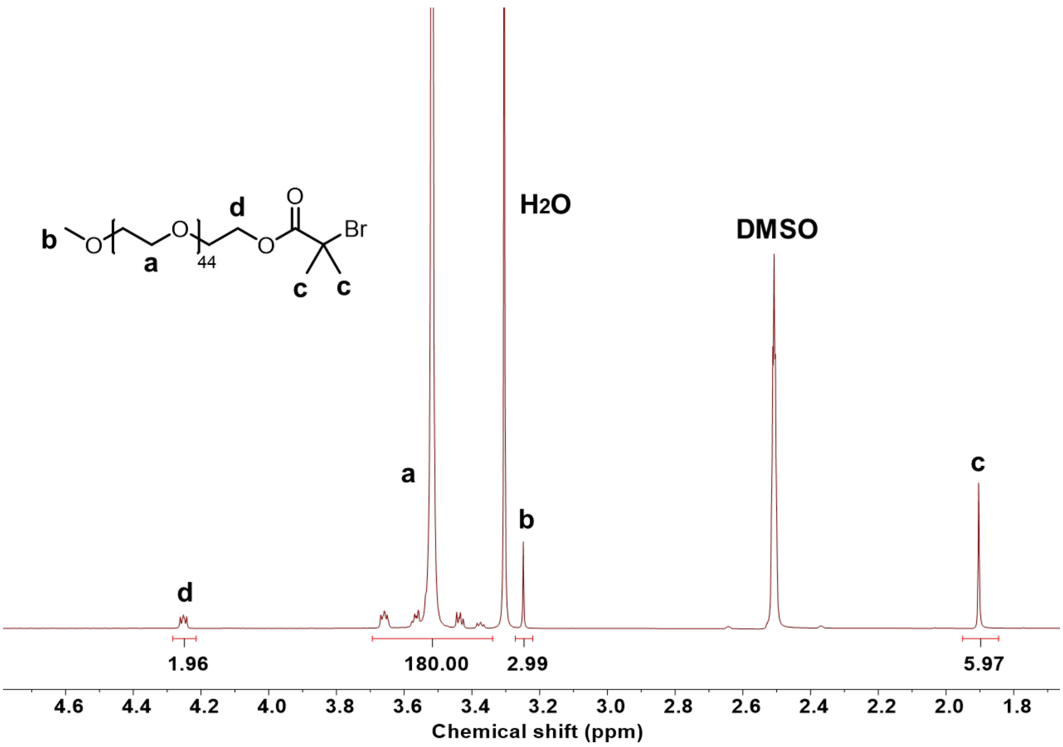

Figure S5. ^{1}H NMR spectrum of CH_3O-PEG Macroinitiator in DMSO-d_6.

Calculation explanation for BCP-A composition:

The intensity of signal “b” from the PEG part is taken as a reference, because of the known total amount of ethylene glycol units (45 units). Since PEG has a symmetric structure, the integral of peak “b” represents 4 H atoms per monomer repeating unit. In contrast, the integrals of the signals “a” referring to the whole hydrophobic block of BCP, represent 2 H atoms each; the integrals of the signals “c”, referring to DMIBMA, represent 2 H atoms each; the integrals of the signals “e+f”, referring to DEAEMA, represent 6 H atoms each; the integrals of the signals “d”, referring to -CH_3, represent 3 H atoms. The integrals of “a”, “b”, “c”, “d” and “e+f” were 221.65, 180, 46.94, 3.27 and 535.63, respectively. Thus, the length of DEAEMA was calculated as about 89 monomer units, whereas the photo crosslinker (DMIBMA) content was found about 24 repeating units. Apart from that, the weight-average molecular weight (M_w) is around 25.0 kDa calculated from the results of ^{1}H NMR spectroscopy.

Calculation explanation for BCP-C composition:

The intensity of signal “b” from the PEG part is taken as a reference, because of the known total amount of ethylene glycol units (45 units). Since PEG has a symmetric structure, the integral of peak “b” represents 4 H atoms per monomer repeating unit. In contrast, the integrals of the signals “a” referring to the whole hydrophobic block of BCP, represent 2 H atoms each; the

integrals of the signals “c”, referring to DMIBMA, represent 2 H atoms each; the integrals of the signals “e+f+g”, referring to DMAEMA and DEAEMA, represent 8 H atoms each; the integrals of the signals “h”, referring to DMAEMA, represent 6 H atoms each; the integrals of the signals “d”, referring to $-CH_3$, represent 3 H atoms. The integrals of “a”, “b”, “c”, “d”, “e+f+g” and “h” were 224.60, 180, 48.80, 3.32, 366.08 and 270.61, respectively. Thus, the length of DMAEMA and DEAEMA were calculated as about 45 monomer units, whereas the photo crosslinker (DMIBMA) content was found about 24 repeating units. Apart from that, the weight-average molecular weight (M_w) is around 24.8 kDa calculated from the results of ^{1}H NMR spectroscopy.

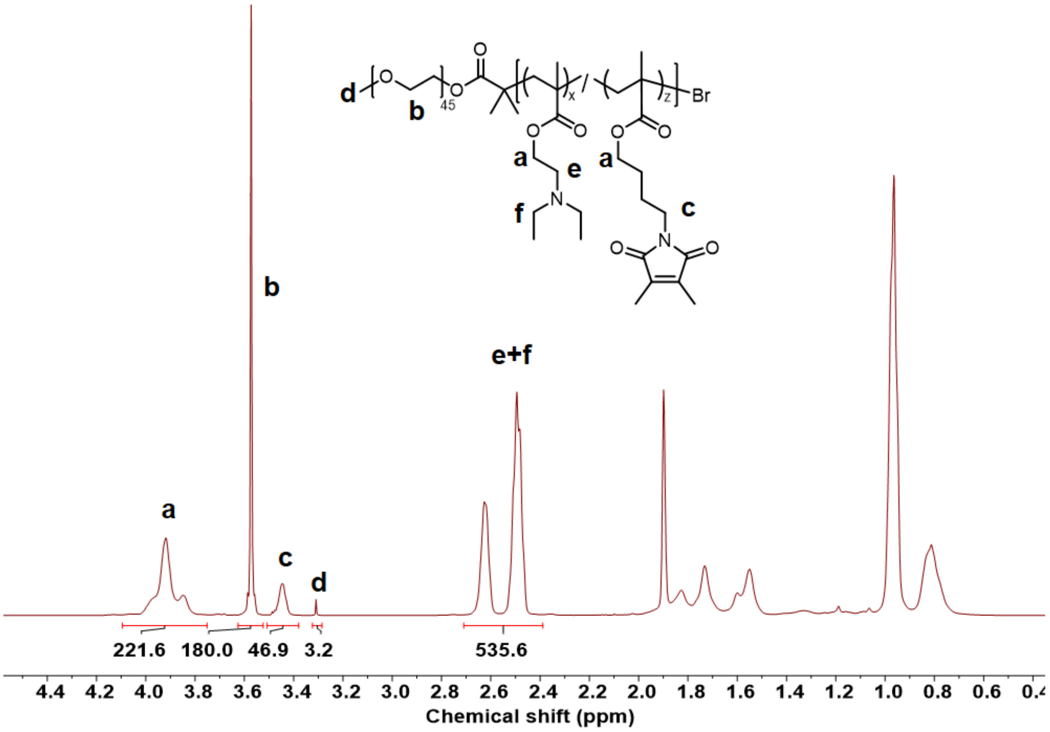

Figure S6. ^{1}H NMR spectrum of PEG$_{45}$-b-(DEAEMA$_{89}$-co-DMIBMA$_{24}$) in $CDCl_3$.

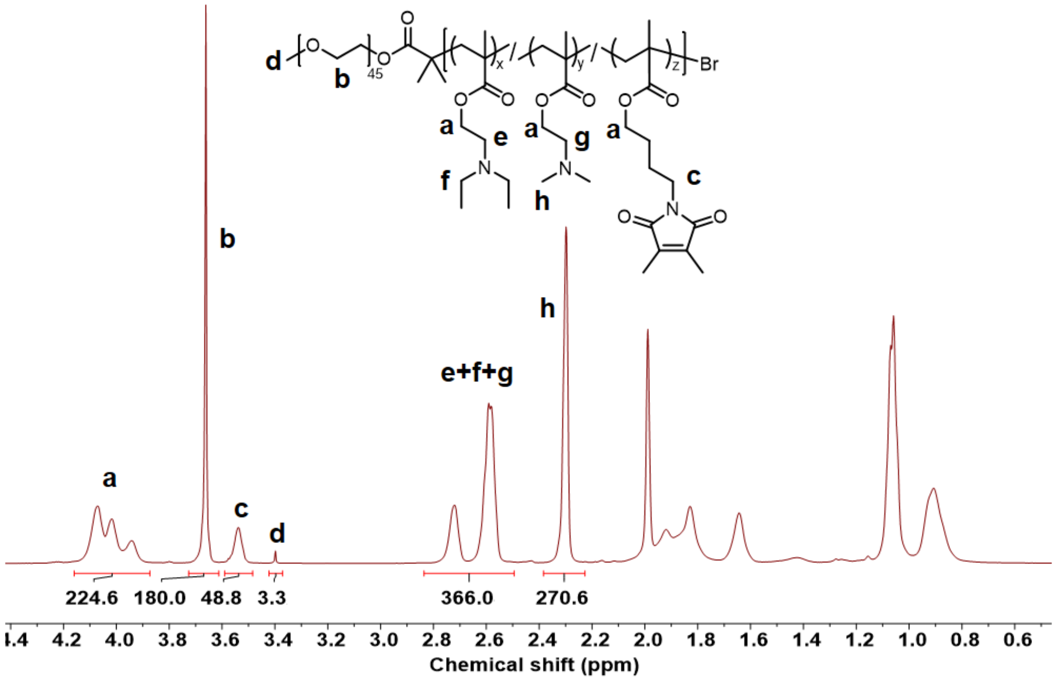

Figure S7. ^{1}H NMR spectrum of PEG$_{45}$-b-(DEAEMA$_{45}$-co-DMAEMA$_{45}$-co-DMIBMA$_{24}$) in $CDCl_3$.

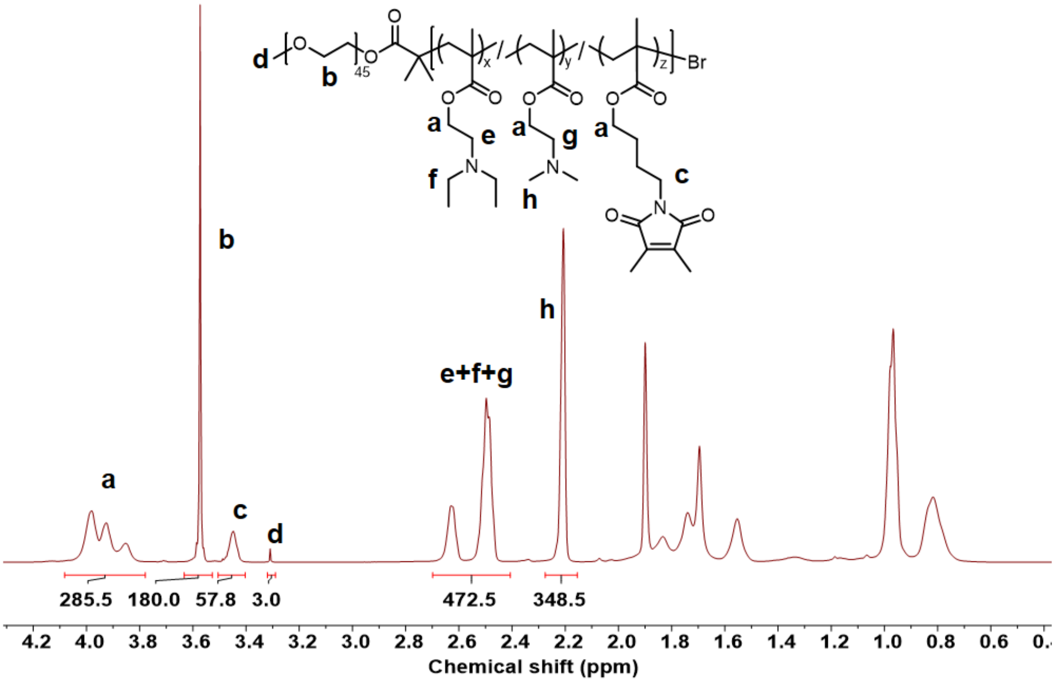

Figure S8. ^{1}H NMR spectrum of PEG$_{45}$-b-(DEAEMA$_{58}$-co-DMAEMA$_{58}$-co-DMIBMA$_{29}$) in $CDCl_3$.

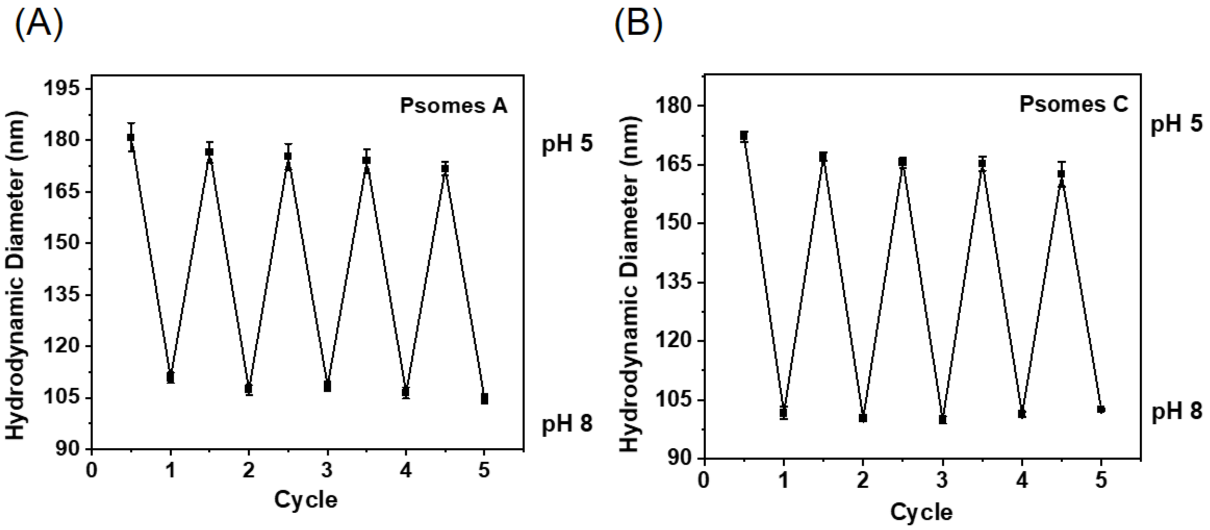

Figure S9. DLS results of Cyclic pH switch of (A) Empty-Psomes A and (B) Empty-Psomes C (1 mg BCP mL^{-1}) crosslinking for 3 min in 10 mM NaCl.

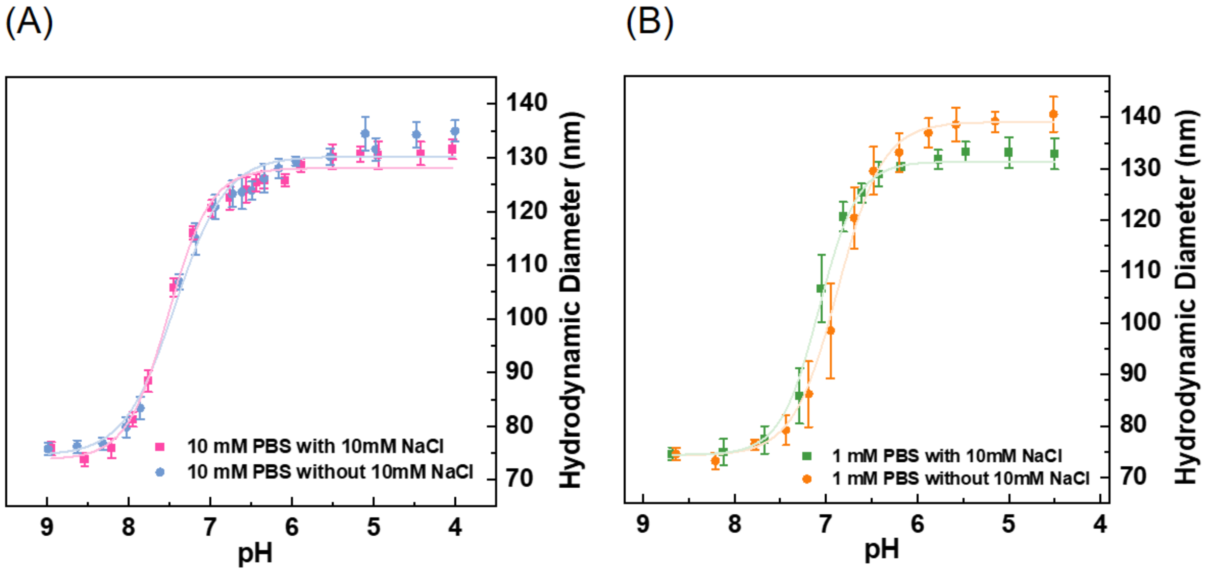

Figure S10. pH-dependent DLS study on Empty-Psomes C in different solutions for the determination of pH* (= turning point for swelling to lower pH and deswelling to higher pH for Empty-Psomes C). Empty-Psomes C (0.5 mg mL^{-1}) in (A) 10 mM PBS buffer with (pH* = 7.51)/without (pH* = 7.43) 10 mM NaCl solution; (B) 1 mM PBS buffer with (pH* = 7.07)/without (pH* = 6.86) 10 mM NaCl solution.

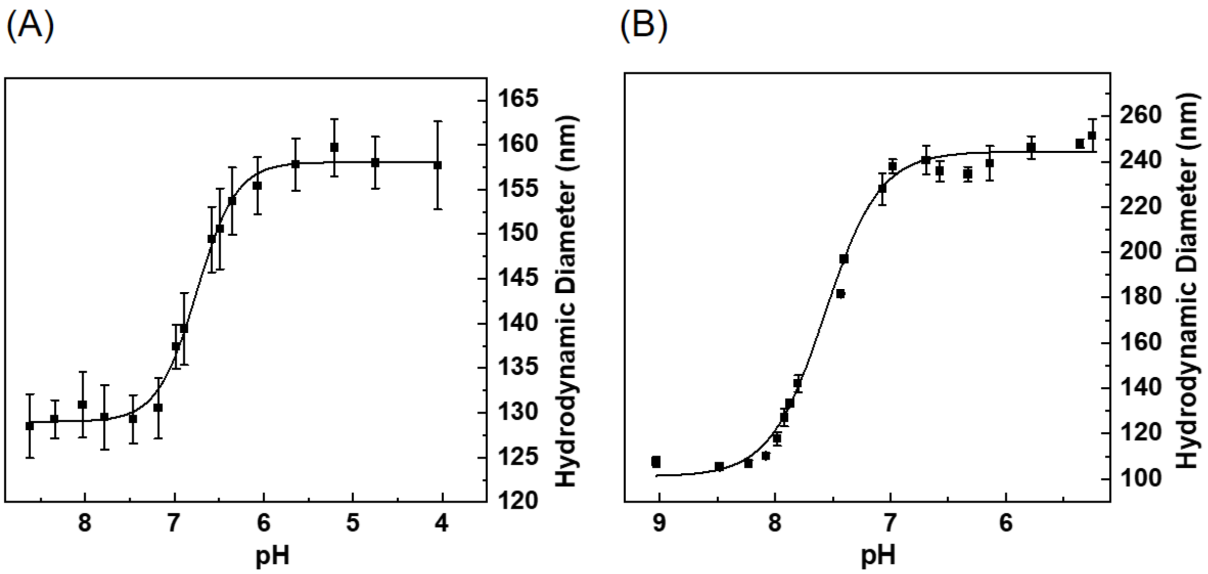

Figure S11. (A) pH-dependent DLS study on Empty-Psomes A and C in 1 mM PBS for the determination of pH* (= turning point for swelling to lower pH and deswelling to higher pH for Empty-Psomes C). (A) Manual titration of Empty-Psomes A (1 mg mL^{-1}, pH* = 6.74) purified by HFF in 1 mM PBS buffer (13.7 mM NaCl, pH 8). (B) Auto titration of Empty-Psomes C (1 mg mL^{-1}, pH* = 7.54) purified by HFF in 1 mM PBS buffer (13.7 mM NaCl, pH 8).

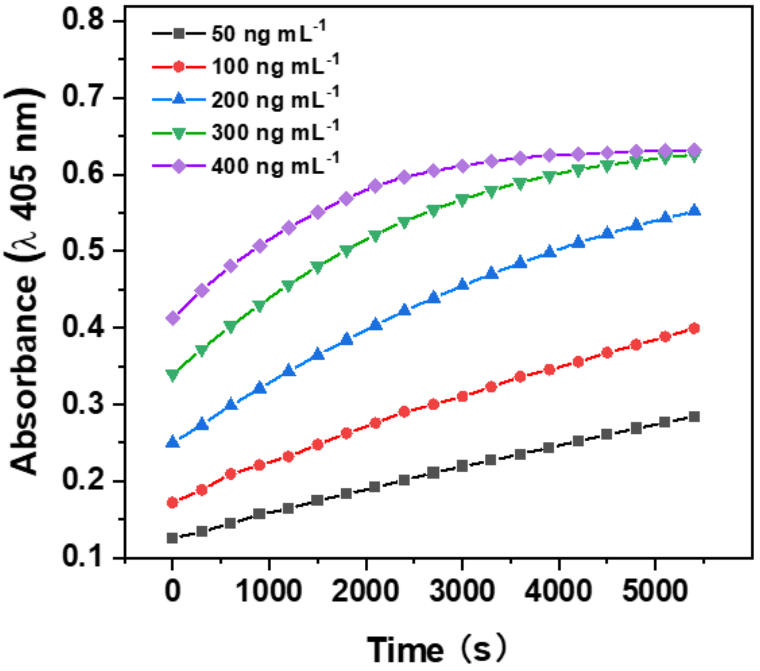

Figure S12. Absorbance of converted substrate in trypsin assay for the determination of trypsin kinetic using different concentrations of trypsin, validated absorbance at λ=405 nm via microplate reader.

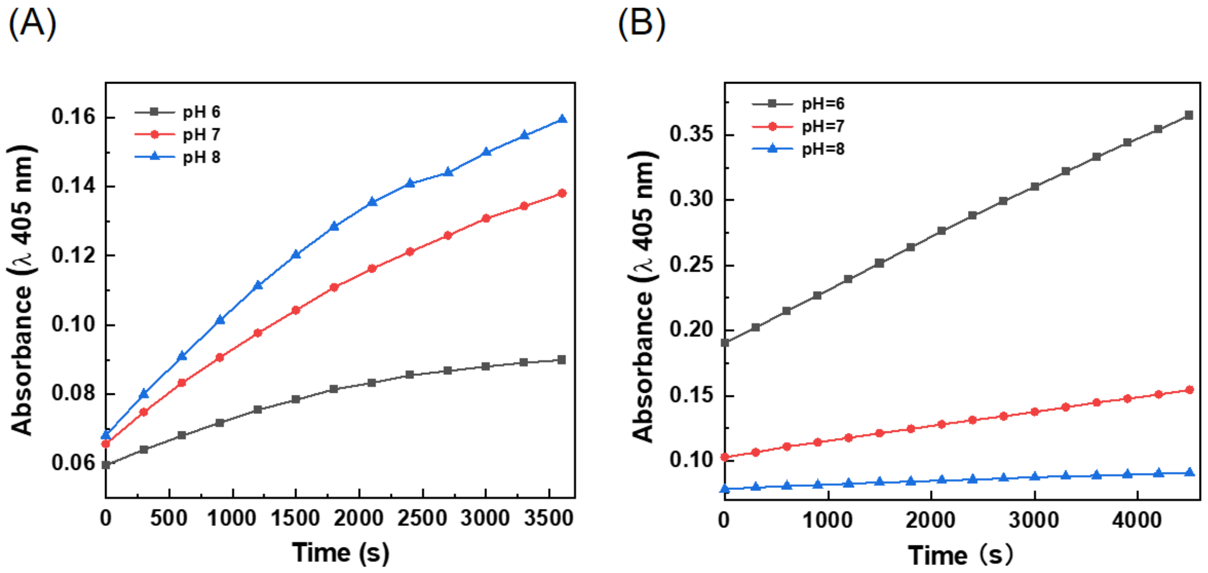

Figure S13. (A) Absorbance of converted substrate in trypsin assay for the determination of pH dependent trypsin activity via microplate reader. Absorbance validated at λ 405 nm for converted substrate in 1 mM PBS buffer at different pH **after 1 h.** (B) Absorbance of converted substrate in trypsin assay for the determination of pH dependent trypsin activity. Absorbance validated at λ 405 nm for converted substrate in 1 mM PBS buffer at different pH **after 1 day**.

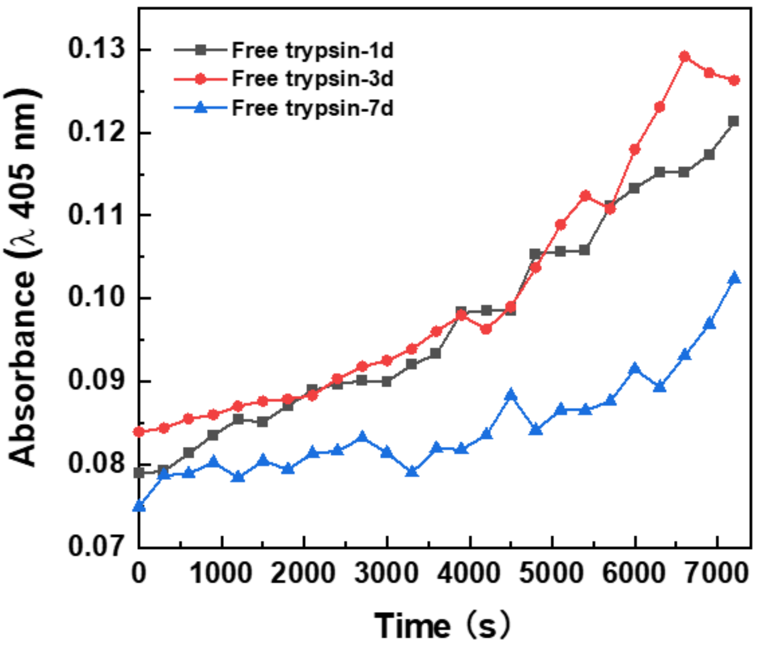

Figure S14. The absorbance of converted substrate at λ 405 nm in trypsin assay for free trypsin in 1 mM PBS buffer, pH 7.4 after 1, 3 and 7 days via microplate reader.

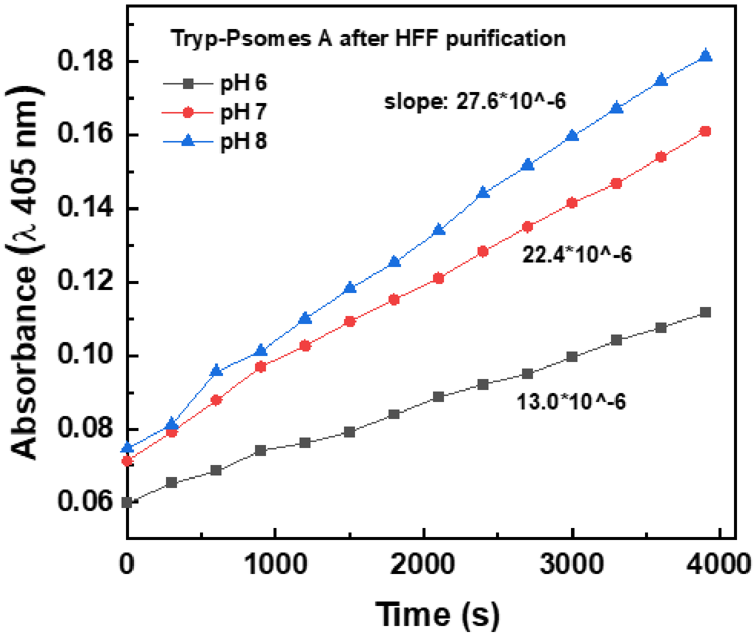

Figure S15. The enzyme activity of Tryp-Psomes A after HFF purification (0.5 mg BCP mL^{-1} in 1 mM PBS buffer) was studied at pH 6, 7 and 8 after 1 h determined by the absorbance of converted substrate in trypsin assay via microplate reader.

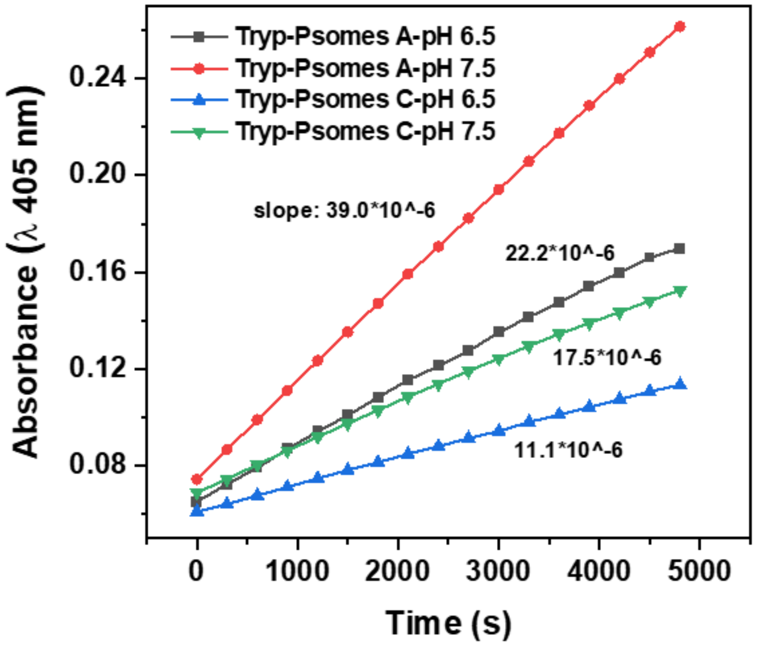

Figure S16. The enzyme activity of purified Tryp-Psomes A and Tryp-Psomes C (0.5 mg BCP mL^{-1} in 1 mM PBS buffer) was studied at pH 6.5 and 7.5 after adjusting the solution pH to 6.5 and 7.5 and keeping the solution for 1 day, respectively, determined by the absorbance of converted substrate in trypsin assay via microplate reader.

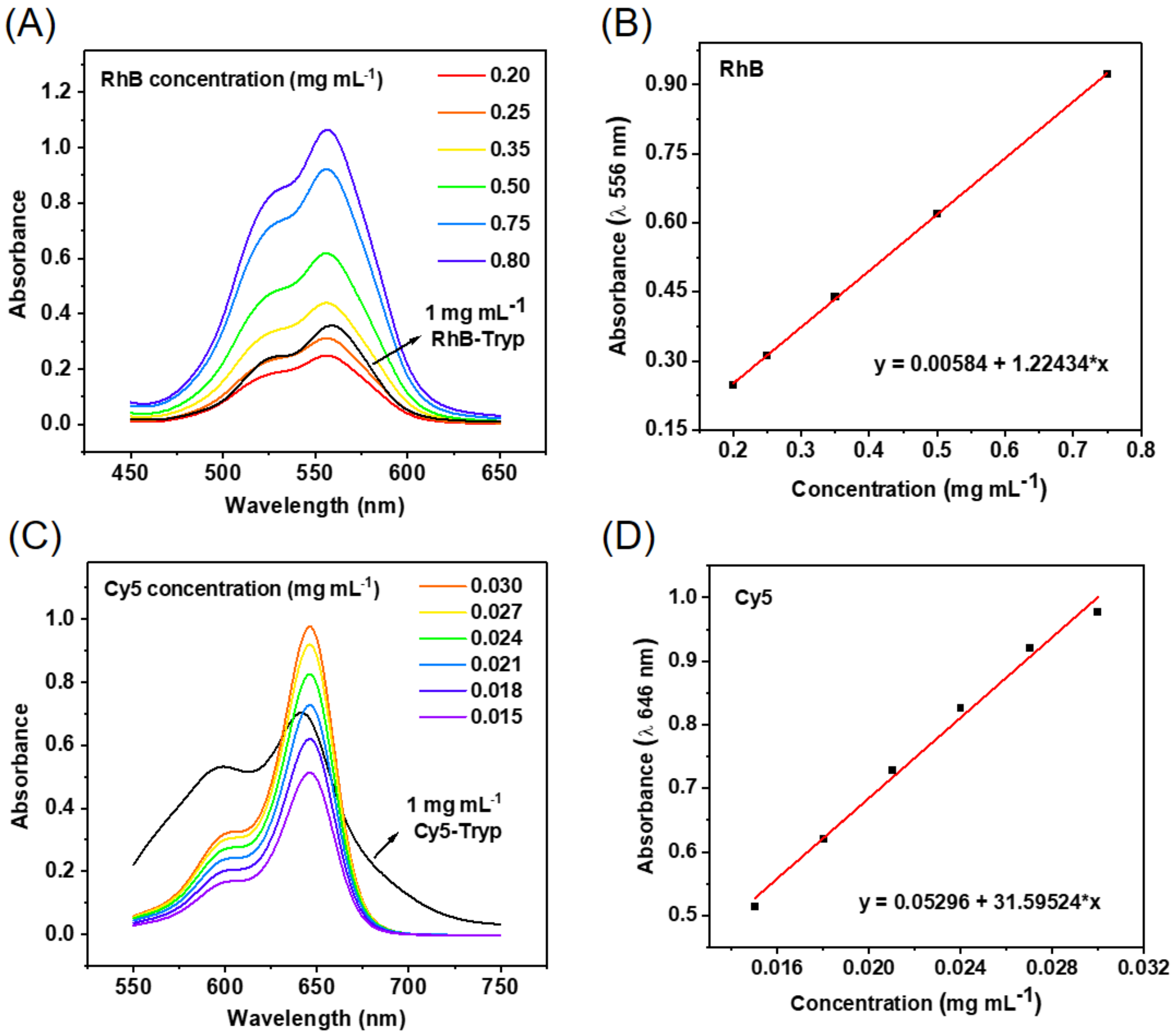

Figure S17. UV spectra and calibration curve of the Dye-Tryp. Every trypsin has been labelled 1.6 RhB for RhB-Tryp, every trypsin has been labelled 0.8 Cy5 for Cy5-Tryp.

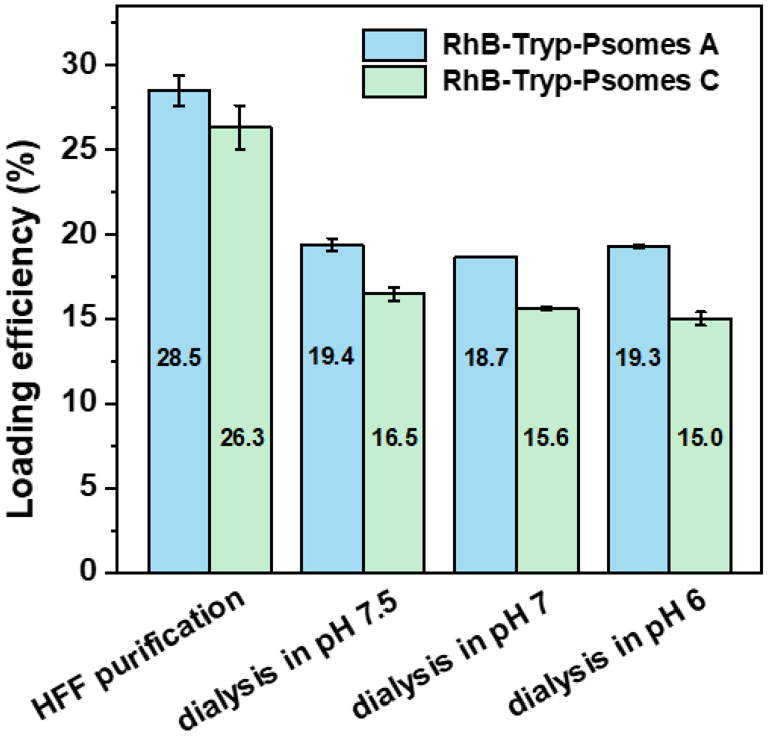

Figure S18. Loading efficiency. Fluorescence intensity of RhB-Tryp-Psomes A and RhB-Tryp-Psomes C after HFF purification (Pressure: 0.11 bar; buffer: 1mM PBS, pH 7.5) and sequential dialysis process at pH 8, 7 and 6 in 1 mM PBS for each 24 h. RhB-Tryp-Psomes A and RhB-Tryp-Psomes C without HFF purification were used as references (100%). Experiments in triplicate. RhB: $\lambda_{ex}/\lambda_{em}$ 543/580 nm.

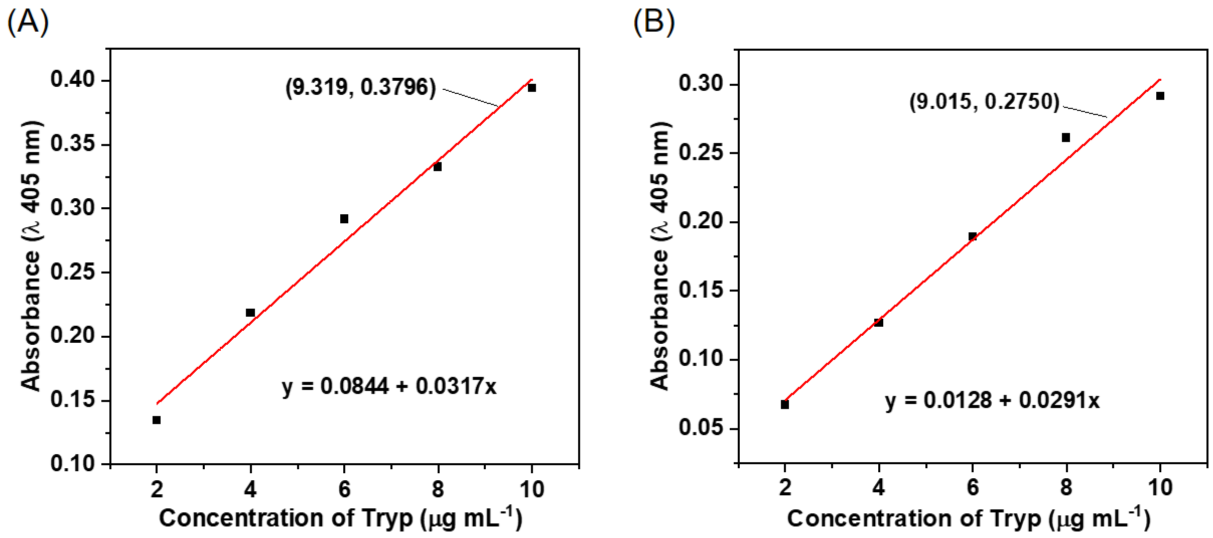

Figure S19. Calibration curve of enzyme activity at pH 7.5 of Tryp-Psomes (0.05 mg mL^{-1} Psomes, 0.01 mg mL^{-1} Tryp) without HFF purification; determined by the absorbance (λ 405 nm) of converted substrate in trypsin assay. The absorbance (λ 405 nm) of (A) Tryp-Psomes A and (B) Tryp-Psomes C (0.5 mg mL^{-1} Psomes, 0.1 mg mL^{-1} Tryp) after HFF purification is 0.3796 and 0.2750, then the loading efficiency of Tryp-Psomes A and Tryp-Psomes C after HFF purification can be calculated as 9.319 μg mL^{-1} ÷ 0.1 mg mL^{-1} × 100% = 9.32% and 9.015 μg mL^{-1} ÷ 0.1 mg mL^{-1} × 100% = 9.02%.

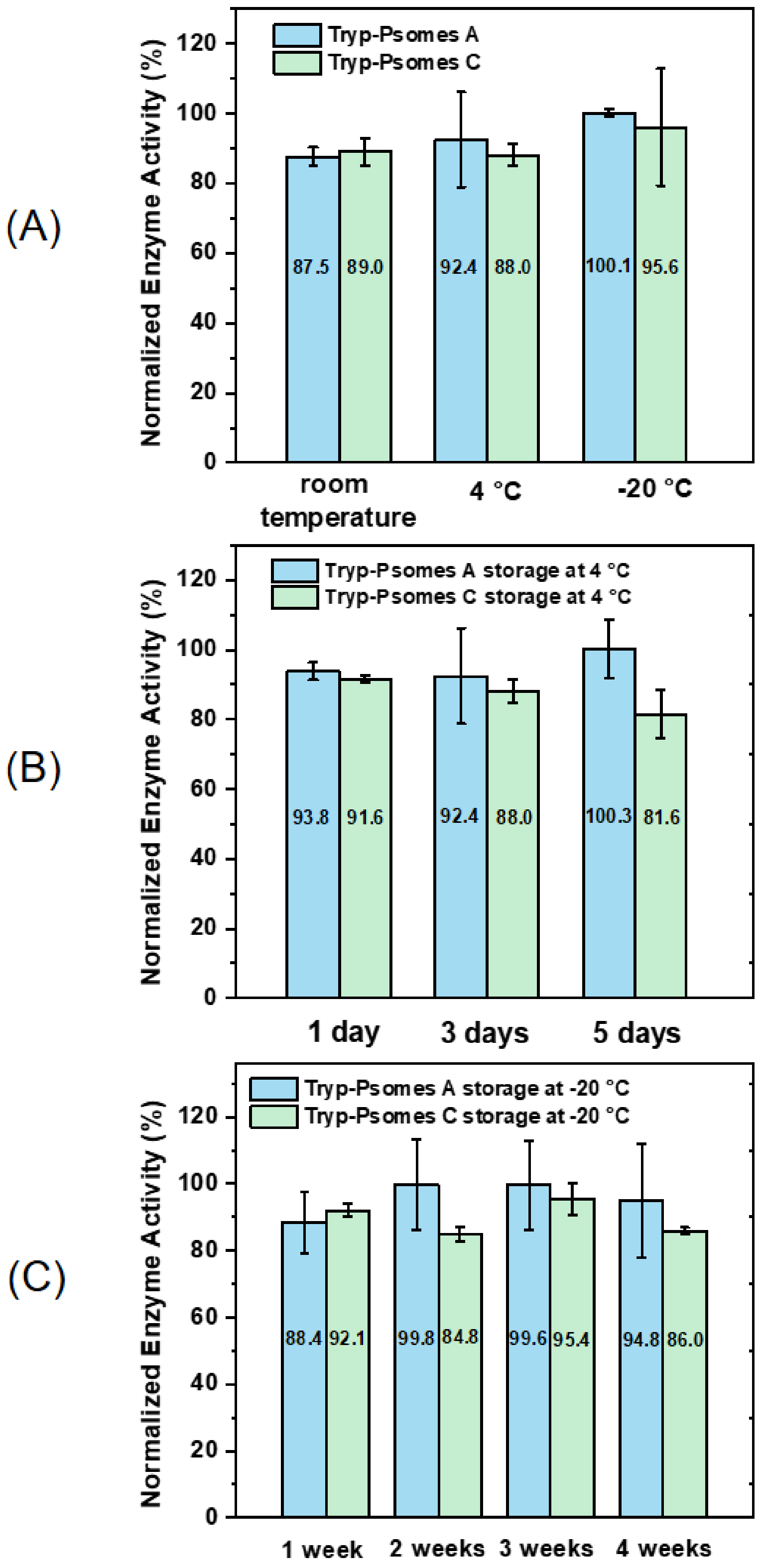

Figure S20. Enzyme activity of (A) purified Tryp-Psomes storage under different condition for three days; (B) Purified Tryp-Psomes storage at 4 °C over time; (C) Purified Tryp-Psomes storage at -20 °C over time. The enzyme activity of fresh HFF purified Tryp-Psomes was used as a reference (100 %). Study of three batches of each Tryp-Psomes sample.

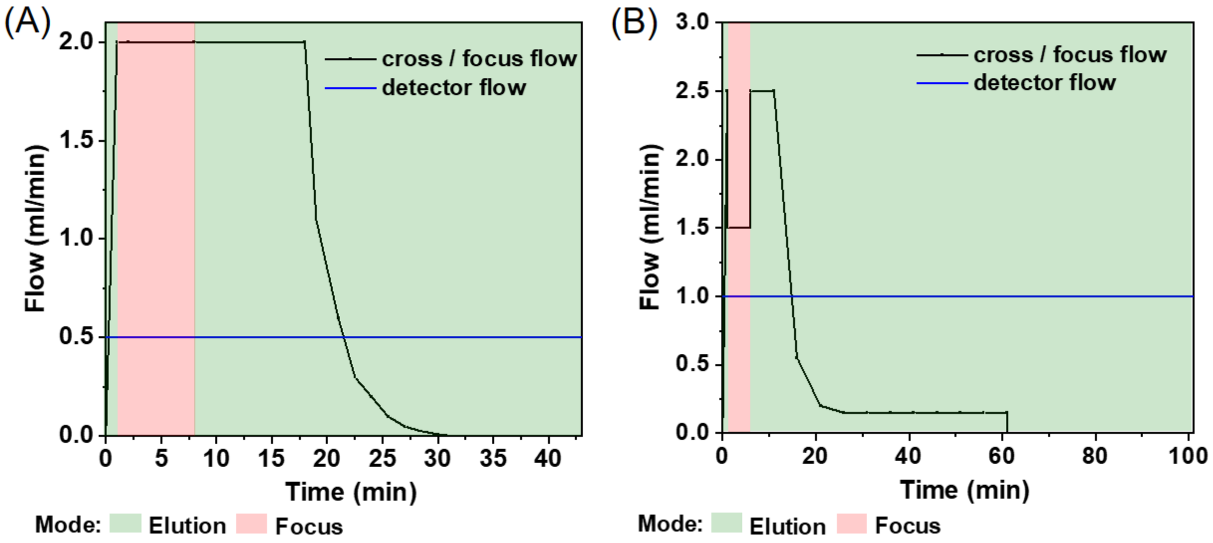

Figure S21. Optimized flow profile for AF4-MALS studies, (A) separation method A for Psomes A and (B) separation method B for Psomes C.

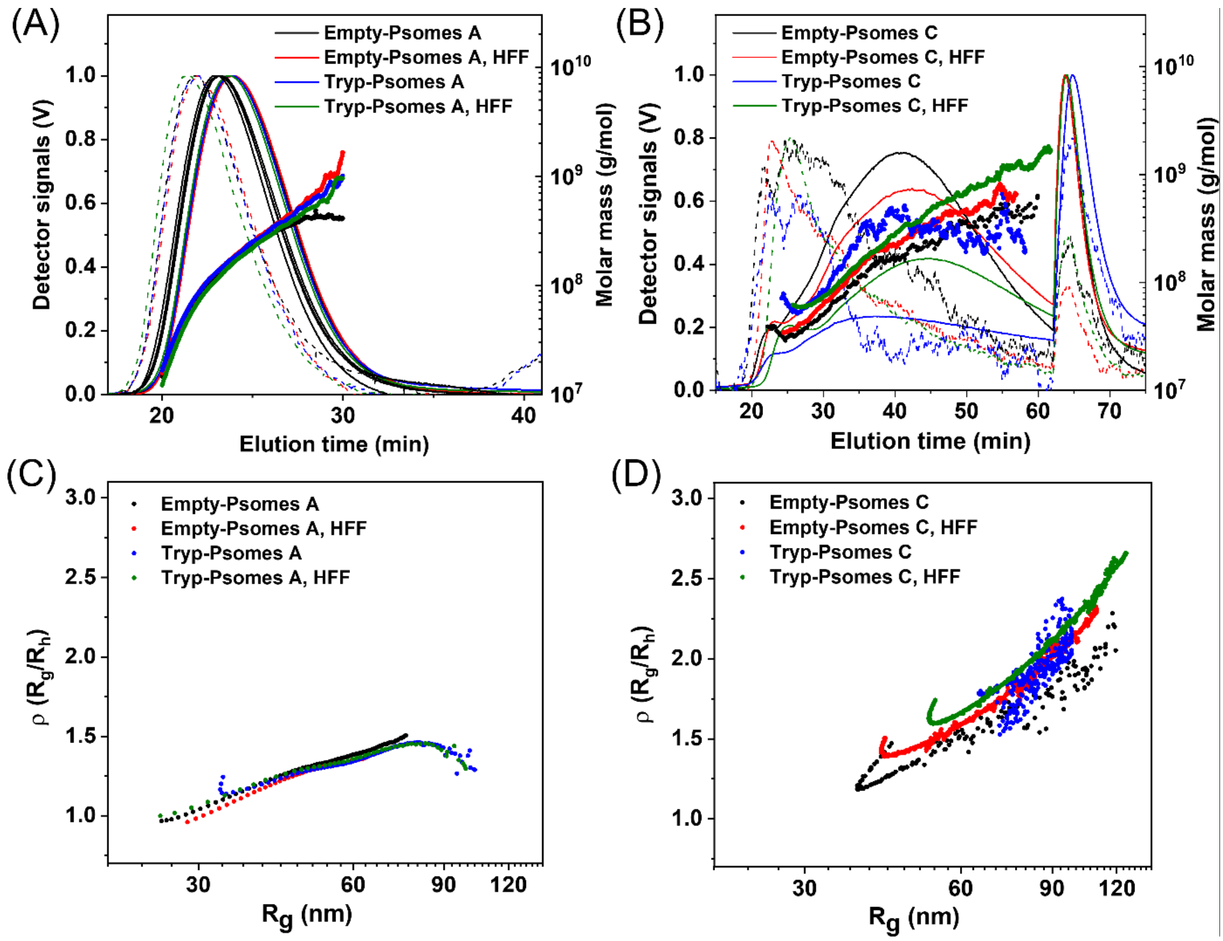

Figure S22. Fractograms of A) Psomes A, separated with Method A and B) Psomes C, separated with Method B, detector signals (LS - solid line, RI - broken line, UV - dotted line) and molar masses (circles) vs elution time; ρ (R_g/R_h) vs R_g of C) Psomes A and D) Psomes C determined by AF4-MALS.

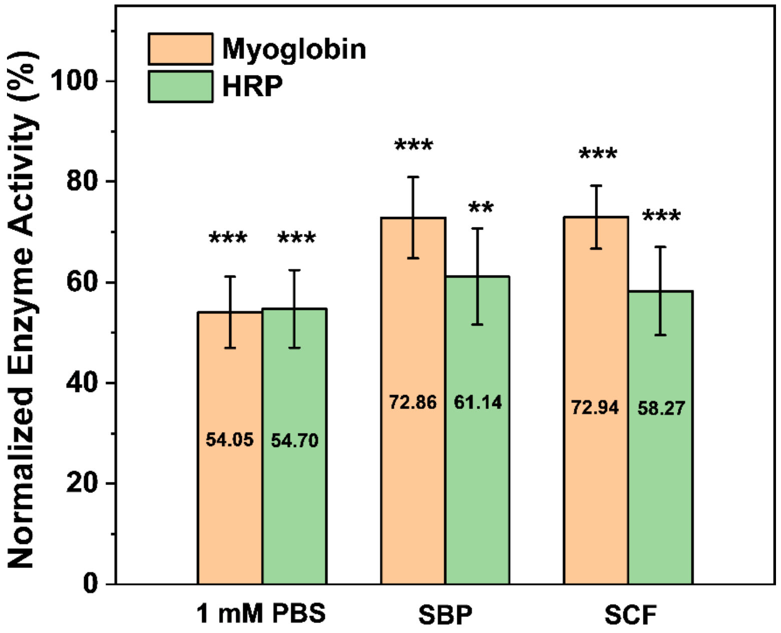

Figure S23. Enzymatic activity of Myo and HRP (0.02 mg mL^{-1}) in the presence of free Tryp (0.04 mg mL^{-1}) in 1 mM PBS at pH 7.5, SBP buffer at pH 7.3 and SCF buffer at pH 7.2, respectively for 24 h (n=3). Normalized free Myo/HRP in 1 mM PBS at pH 7.5, SBP buffer at pH 7.3 and SCF buffer at pH 7.2, respectively for 24 h enzyme activity as 100 %, which is carried out at room temperature. Statistical significance was indicated with (*) for $p < 0.05$, (**) for $p < 0.01$, and (***) for $p < 0.001$, respectively.

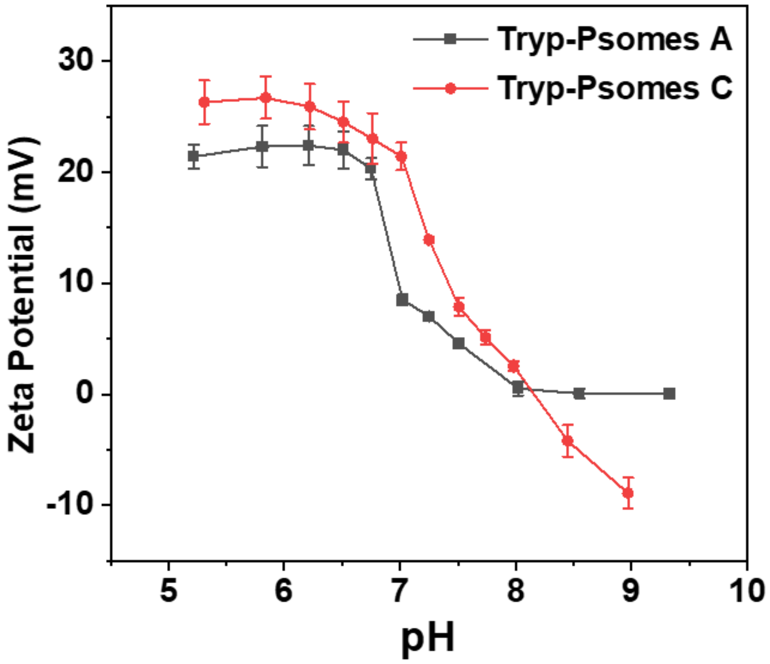

Figure S24. The manual titration curve of Tryp-Psomes A and Tryp-Psomes C (1 mg mL^{-1}) purified by HFF in 1 mM PBS buffer (13.7 mM NaCl, pH 7.5).

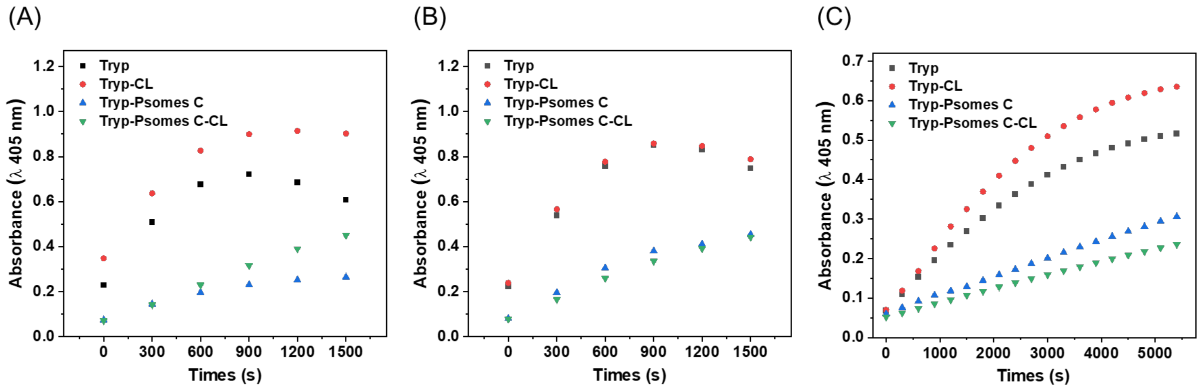

Figure S25. The enzyme activity of free trypsin and Tryp-Psomes C before and after crosslinking (CL), trypsin concentration of (A) and (B) is 2 μg mL^{-1}, of (C) is 0.2 μg mL^{-1}. Psomes solution used as unpurified samples to have the same amount of Tryp in each sample. Experimental details in SI.

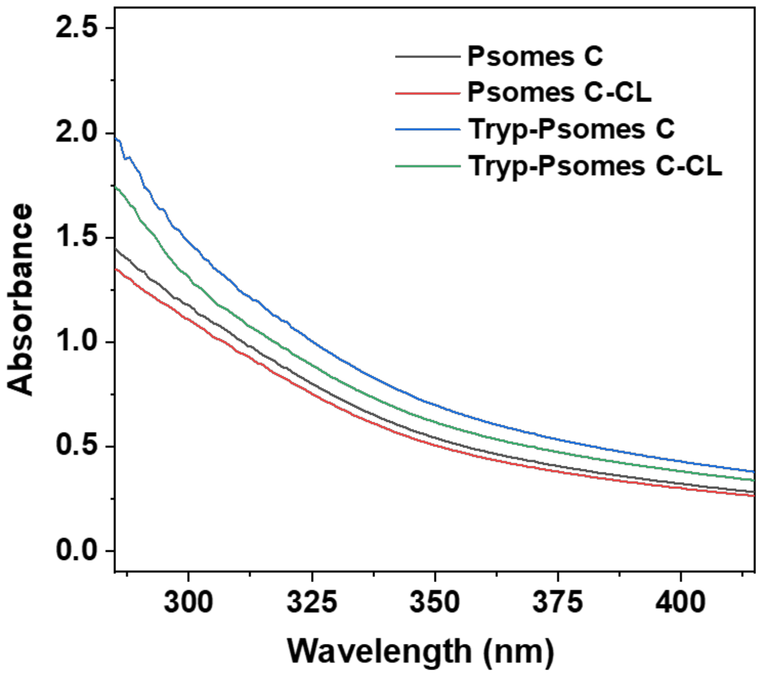

Figure S26. The UV-Vis spectra of Empty-Psomes C (1 mg BCP-C mL^{-1}) and Tryp-Psomes C (1 mg BCP-C mL^{-1} and 0.2 mg Tryp mL^{-1}) before and after crosslinking (CL). There is no chance to validate the crosslinking efficiency due to scattering of laser light and low concentration of crosslinker in the slightly swollen membrane of Tryp-Psomes C under physiological pH.

Table S1. Specifications of block copolymer synthesized by ATRP

Code	Composition	M_w (g mol^{-1})^a	M_n (g mol^{-1})^a	$Đ$ (M_w/M_n)^a	M_n (g mol^{-1})^b
**BCP-A**	PEG_{45}-b-($DEAEMA_{89}$-co-$DMIBMA_{24}$)	49400	86700	1.75	25000
**BCP-C**	PEG_{45}-b-($DEAEMA_{45}$-co-$DMAEMA_{45}$-co-$DMIBMA_{24}$)	35200	43200	1.22	24800

^{a} Molar mass distribution is determined by SEC. ^{b} Molecular weight is calculated by ^{1}H NMR.

Table S2. The parameter of the Omnicure device before and after change the UV lamps using the new conduits.

Using time	Intensity (W)	Irradiance (W cm^{-2})	Crosslinking time
115 hours	3.08	15.6	180 s (using the old conduit)
0 h	5.33	27.1	40 s

Remark: Not only we did replace the UV lamp with the new one during this study, we also replaced the conduit for light channel with the new one. The damaged conduit also affects the efficiency of photo-crosslinking.

Table S3. The hydrodynamic diameter of Empty-Psomes C (1 mg BCP-C mL^{-1}) and Tryp-Psomes C (1 mg BCP-C mL^{-1} and 0.2 mg Tryp mL^{-1}) before and after crosslinking (CL).

Samples	(nm)	STD	PDI
Empty-Psomes C	95.1	0.76	0.13
Empty-Psomes C-CL	96.1	0.86	0.099
Tryp-Psomes C	109.4	0.95	0.13
Tryp-Psomes C-CL	111.4	1.054	0.111

Table S4. Monitor the size of the free trypsin dissolve in 1 mM PBS buffer, pH 7.4 in 1, 2 and 3 days

Samples	Size (nm)	PDI	Zeta (mV)
Tryp -1 day	7.515	0.331	6.08
Tryp -2 days	1,814	0.884	1.29
Tryp -3 days	1,277	0.735	-1.38

Table S5. Results of AF4-MALS studies in 1 mM PBS buffer

Samples	M_n^* (kg mol^{-1})	M_w^* (kg mol^{-1})	Đ (M_w/M_n)	R_g (nm)	R_h (nm)
Empty-Psomes A	55,700	123,000	2.21	62.1	43.7
Empty-Psomes A, HFF	68,500	146,000	2.13	64.6	46.6
Tryp-Psomes A	61,400	139,000	2.26	62.3	45.4
Tryp-Psomes A, HFF	39,500	129,000	3.16	66.4	46.7
Empty-Psomes C	64,400	215,000	3.34	111	47.9
Empty-Psomes C, HFF	68,100	237,000	3.48	113	44.3
Tryp-Psomes C	193,000	557,000	2.89	128	46.6
Tryp-Psomes C, HFF	120,000	442,000	3.73	136	44.4

* dn/dc= 0.183 ml g^{-1}

Table S6. Zeta potential of enzymes dissolved in different solutions at different pH values

Solvent	Condition	Zeta potential (mV)		
		Tryp	HRP	Myo
Milli-Q water	pH 6.5	20.5 ±1.27	-8.79 ±0.49	-2.82 ±0.21
	pH 7.5	17.1 ± 1.36	-11.3 ± 0.81	-27.3 ±2.94
1 mM PBS	pH 6.5	4.11 ±0.39	-4.40 ± 0.28	-15.6 ± 0.75
	pH 7.5	0.65 ±0.64	-6.20 ±0.13	-9.77 ±1.31

Table S7. Amino acid properties in different pH

Amino acid	Hydrophobic	Neutral	Hydrophilic
pH 2	L, I, F, W, V, M, C, Y, A	T, E, G, S, Q, D	R, K, N, H, P
pH 7	F, I, W, L, V, M, Y, C, A	T, H, G, S, Q	R, K, N, E, P, D

Reference from: https://www.sigmaaldrich.com/DE/en/technical-documents/technical-article/protein-biology/protein-structural-analysis/amino-acid-reference-chart

Table S8. Properties of Myo and HRP

Protein	Myoglobin		Horseradish peroxidase	
Source	from horse skeletal muscle		isolated from horseradish roots (Amoracia rusticana)	
Isoelectric point	7.3 (major component) and 6.8 (minor component)		Isozymes range from 3.0 - 9.0	
Molecular weight	17.6 kDa		approx. 44 kDa	
Number of amino acid residues	153		includes the polypeptide chain (33,890 Daltons), hemin plus Ca^{2+} (approx. 700 Daltons), and carbohydrate (9400 Daltons).	
	pH 2	pH 7	pH 2	pH 7
Hydrophobic fragment	60	60	130	130
Neutral fragment	55	42	99	76
Hydrophilic fragment	39	52	77	100
	Hydrophobic		Hydrophobic	
Protein sequence	MGLSDGEWQLVLNVWGKV<br>EADIPGHGQEVLIRLFKGHPE<br>TLEKFDKFKHLKSEDEMKAS<br>EDLKKHGATVLTALGGILKK<br>KGHHEAEIKPLAQSHATKHK<br>IPVKYLEFISECIIQVLQSKHP<br>GDFGADAQGAMNKALELFR<br>KDMASNYKELGFQG<br>154 residues, mass: 17184 Da		QLRPDFYSRTCPSVFNIIKNVIVDE<br>LQTDPRIAASILRLHFHDCFVRGC<br>DASILLDTSKSFRTEKDAAPNVNS<br>ARGFNVIDRMKTALERACPRTVS<br>CADILTIASQISVLLSGGPSWAVPL<br>GRRDSVEAFFDLANTALPSPFFTL<br>AQLKKAFADVGLNRPSDLVALSG<br>GHTFGRARCLFVTARLYNFNGTN<br>RPDPTLNPSYLADLRRLCPRNGN<br>GTVLVNFDVMTPNTFDNQFYTNL<br>RNGKGLIQSDQELFSTPGADTIPL<br>VNLYSSNTLSFFGAFADAMIRMG<br>NLRPLTGTQGEIRQNCRVVNSR<br>306 residues, mass: 33721 Da	

Protein sequence reference from: NCBI, the National Center for Biotechnology Information advances science and health by providing access to biomedical and genomic information. https://www.ncbi.nlm.nih.gov

Table S9. The hydrodynamic diameter (D_h) of HRP and Myo in different buffer solutions after 1 h dissolving and filtration with 0.2 µm pore size.[a]

**Hydrodynamic diameter in different buffer solution**	**HRP**			**Myo**		
	0.2 mg mL^{-1}					
	Size (nm)	STD	PDI	Size (nm)	STD	PDI
1 mM PBS buffer, pH 7.5	10.1	0.57	0.38	10.1	0.95	0.43
SBP buffer, pH 7.3	10.2	0.74	0.28	8.5	0.39	0.41
SCF buffer, pH 7.2	10.0	0.33	0.31	8.2	0.38	0.33

[a]Myo and HRP outline some slight aggregation characteristics. Thus D_h does not reflect the original smaller diameters from literature in suited solvent for both.

3. Reference

[1] J. Roovers, *Branched polymers II, Vol. 143*, Springer, **2003**.

[2] W. Burchard, *Branched polymers II* **1999**, 113-194.

[3] J. Gaitzsch, D. Appelhans, L. Wang, G. Battaglia, B. Voit, *Angewandte Chemie International Edition* **2012**, *51*, 4448-4451.

[4] H. Gumz, T. H. Lai, B. Voit, D. Appelhans, *Polymer Chemistry* **2017**, *8*, 2904-2908.

[5] S. Moreno, P. Sharan, J. Engelke, H. Gumz, S. Boye, U. Oertel, P. Wang, S. Banerjee, R. Klajn, B. Voit, *Small* **2020**, *16*, 2002135.

[6] S. Moreno, S. Boye, A. Lederer, A. Falanga, S. Galdiero, S. Lecommandoux, B. Voit, D. Appelhans, *Biomacromolecules* **2020**, *21*, 5162-5172.

[7] M. R. Marques, R. Loebenberg, M. Almukainzi, *Dissolution Technol* **2011**, *18*, 15-28.

[8] E. McGale, I. Pye, C. Stonier, E. Hutchinson, G. Aber, *Journal of neurochemistry* **1977**, *29*, 291-297.
